# IFITM2 Modulates Endocytosis Maintaining Neural Stem Cells in Developing Neocortex

**DOI:** 10.1002/advs.202501593

**Published:** 2025-03-07

**Authors:** Yuqing Lv, Wenzheng Zou, Lin Li, Shukui Zhang, Jiaqi Liang, Jiali Pu, Jianwei Jiao

**Affiliations:** ^1^ Key Laboratory of Organ Regeneration and Reconstruction Chinese Academy of Science Beijing 100101 China; ^2^ University of Chinese Academy of Sciences Beijing 100049 China; ^3^ Beijing Institute for Stem Cell and Regenerative Medicine Institute for Stem Cell and Regeneration Chinese Academy of Sciences Beijing 100101 China; ^4^ Affiliated Hospital of Guangdong Medical University & Key Laboratory of Zebrafish Model for Development and Disease of Guangdong Medical University Zhanjiang 523710 China; ^5^ Department of Neurology Second Affiliated Hospital Zhejiang University School of Medicine Hangzhou 310009 China; ^6^ Co‐Innovation Center of Neuroregeneration Nantong University Nantong 226001 China

**Keywords:** brain development, endocytosis, IFITM2, neural stem cell, phosphoinositide

## Abstract

Brain development is orchestrated by a complex interplay of genetic and environmental signals, with endocytosis serving as a pivotal process in integrating extracellular cues. However, the specific role of endocytosis in neurogenesis remains unclear. We uncover a critical function of the interferon‐induced transmembrane protein, IFITM2, essential for endocytic processes in radial glial cells (RGCs). IFITM2 is highly expressed near the ventricular surface in the developing brain. Loss of IFITM2 impairs endosome formation and disrupts RGC maintenance. Mechanistically, we confirmed that the YXXø endocytic motif on IFITM2 is essential for its subcellular localization, with mutations in this motif reducing endocytic vesicles. Additionally, the K82 and K87 residues of IFITM2 interact with phosphoinositides to promote endocytic vesicle formation. Polarized localization of phosphatidylinositol 3,4‐bisphosphate (PI(3,4)P2) on the ventricular side suggests its role in vesicle formation. IFITM2 deficiency also leads to reduced phosphorylation of AKT and GSK3β. These findings highlight the essential role of IFITM2 in regulating endocytosis in RGCs, which is critical for maintaining neural stem cells and proper brain development, offering new insights into the connection between cellular signaling and neurogenesis in both mouse and human models.

## Graphical abstract

This study reveals IFITM2 as a key regulator of endocytosis in radial glial cells (RGCs) during brain development. IFITM2's YXXø motif and phosphoinositide‐binding residues (K82/K87) are essential for endosome formation. IFITM2 deficiency disrupts the embryonic cortical neurogenesis, revealing a conserved mechanism linking endocytic dynamics to neural stem cell homeostasis in mammals.

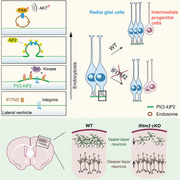

## 1 Introduction

The cerebral cortex, responsible for high‐order cognitive and sensorimotor processing, evolves through complex mechanisms involving the proliferation and differentiation of neural progenitor cells.^[^ ^1^ ^]^ These dynamic processes are regulated by intrinsic genetic programs and various environmental cues. Recent evidence has underscored the critical role of endocytosis, particularly the asymmetric inheritance of endolysosomes—organelles formed from the fusion of endosomes and lysosomes—in determining the fate of radial glial progenitor cells (RGCs).^[^ ^2^ ^]^ This pivotal role highlights the broader importance of the endocytic pathway in neocortical development. Further, the dynamics of endosomes are increasingly recognized as a conserved evolutionary strategy for cortical neurogenesis, evidenced by similar mechanisms in Drosophila, where endosome behavior affects Notch signaling during neural progenitor cell division.^[^ ^3^ ^]^ Despite these insights, the detailed molecular and mechanistic aspects of endocytosis in neocortical development remain poorly defined.

This study seeks to delineate the role of interferon‐induced transmembrane protein 2 (IFITM2) in the developing mammalian neocortex. Known primarily for its antiviral defenses, IFITM2 inhibits viral infection by altering membrane curvature, impacting the stages of cellular entry and viral fusion.^[^ ^4^ ^]^ Intriguingly, elevated expressions of IFITM2 in RGCs suggest its potential beyond immunological functions, possibly impacting neurodevelopmental processes. This notion is supported by analogous functions observed in IFITM3, which has been implicated in modulating neurodegenerative^[^ ^5^ ^]^ and oncogenic pathways.^[^ ^6^ ^]^ Our findings reveal a novel function of IFITM2 in regulating endocytosis in the developing brain and elucidate how IFITM2 interacts with polarized PI(3,4)P2 signaling in RGCs, offering fresh insights into the molecular orchestration of brain development.

## 2 Results

### 2.1 Spatiotemporal Consistency of Endocytosis and IFITM2 Expression in Mouse and Human Developing Neocortex

**Figure 1:**
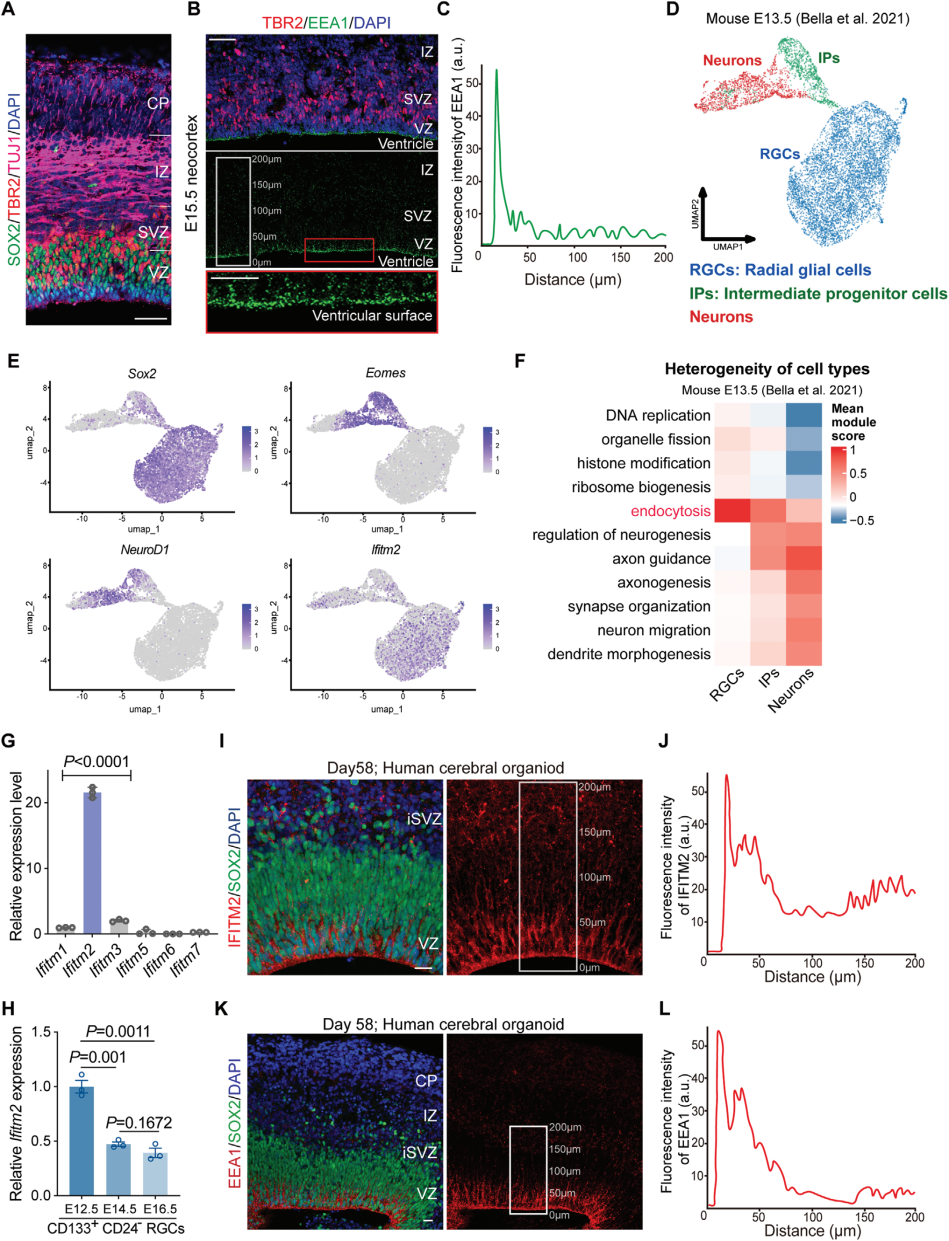
Endocytosis and IFITM2 expression show spatiotemporal consistency in the developing mouse neocortex and human cerebral organoids. A) Representative image of E15.5 neocortex stained for SOX2 (green), TBR2 (red), TUJ1 (magenta), and nuclei with DAPI (blue). Scale bar, 50 µm. VZ, ventricular zone; SVZ, subventricular zone; IZ, intermediate zone; CP, cortical plate. B,C) Representative images of E15.5 neocortex stained for EEA1 (early endosome antigen 1, green), TBR2 (red), and nuclei with DAPI (blue). The red rectangle indicates the ventricular surface region of the neocortex, which is displayed at higher magnification at the bottom. The white rectangle marks the region where EEA1 fluorescence intensity was quantified in panel (C). Scale bars in (B), 50 µm (above) and 25 µm (below). D) UMAP visualization of the scRNA‐seq data from mouse E13.5 somatosensory neocortex.^[^ ^11a^ ^]^ Unsupervised Leiden clustering and classification of cells after integration with Harmony algorithm. E) UMAP visualization of expression levels (normalized) of marker genes for radial glial cells (*Sox2*), intermediate progenitors (*Eomes*), newborn neurons (*Neurod1*), and *Ifitm2*. F) Heatmap showing processes enriched in different cell types from scRNA‐seq data for E13.5 mouse neocortex. G) Q‐PCR analysis of *Ifitm* family genes expression from the E11.5 mouse brains (*n =* 3 independent experiments). All Ct values were normalized to *Gapdh* control. Data are shown as mean ± s.e.m. One‐way ANOVA followed by Tukey's post‐hoc test was used. H) Q‐PCR analysis of *Ifitm2* mRNA levels in CD133^+^CD24^−^ RGCs at E12.5, E14.5, and E16.5 stages (*n* = 3 independent experiments). All Ct values were normalized to *Gapdh* control. Data are shown as mean ± s.e.m. Two‐tailed unpaired Students’ *t*‐test was used. I,J) Representative images of day 58 human cerebral organoid immunostaining with IFITM2 (red), SOX2 (green), and nuclei with DAPI (blue). Scale bar, 20 µm. The white rectangle marks the region where IFITM2 fluorescence intensity was quantified in (J).K,L) Representative images of day 58 human cerebral organoid immunostaining with EEA1 (red), SOX2 (green), and DAPI (blue). Scale bar, 20µm. The white rectangle marks the region where EEA1 fluorescence intensity was quantified in (L).

In the developing mouse neocortex, we used immunostaining to label radial glial progenitor cells (RGCs), intermediate progenitor cells (IPs), and differentiated neurons with SOX2, TBR2, and TUJ1, respectively (**Figure** **1**A). The ventricular zone (VZ) is predominantly populated by RGCs, the sub‐ventricular zone (SVZ) by IPs, and the intermediate zone (IZ) and cortical plate (CP) by post‐mitotic neurons. To assess endocytosis in the neocortex, we stained early endosomes with EEA1 on embryonic day (E) 15.5, revealing a high density of early endosomes near the lateral ventricles, indicating robust endocytic activity in the RGCs near the ventricular surface (Figure 1B,C). We tracked early endosome numbers on the ventricular surface at E11.5, E13.5, and E15.5, observing a progressive decline from early to late neurogenesis, suggesting a reduction in the endocytic capacity of RGCs over time (Figure S1A–C, Supporting Information). In addition, EEA1 signaling exhibits regional specificity near the ventricles. Compared to the ganglionic eminence region adjacent to the ventricles, the neocortex shows a higher density of endocytic signals (Figure S1A–C, Supporting Information). These findings suggest that endocytosis in the developing neocortex is spatially regulated, with RGCs near the ventricular surface displaying robust endocytic activity, which gradually decreases as neurogenesis progresses. This regional variation in endocytic activity might reflect distinct functional roles of endocytosis in different progenitor populations during cortical development.

Given the limited understanding of the molecular basis of endocytosis in RGCs, we analyzed single‐cell RNA transcriptional profiles from the E13.5 developing somatosensory cortex.^[^ ^7^ ^]^ Initial identification of cell types in the neocortex highlighted a correlation between RGCs and endocytic biological processes (Figure 1D–F). Notably, our analysis identified a high expression of IFITM2 in RGCs (Figure 1E). Moreover, IFITM2 expression was significantly higher than that of IFITM1 and IFITM3 within the IFITM family at E11.5, with negligible expression of other family members (Figure 1G). Flow cytometry of isolated CD133^+^CD24^−^ RGCs further confirmed high and progressively decreasing IFITM2 expression from early to late neurogenesis (Figure 1H; Figure S1D, Supporting Information). Next, we stained human cerebral organoid slices for IFITM2. We observed a dense presence of IFITM2 near ventricle‐like structures (Figure 1I,J; Figure S1E, Supporting Information), which was similar to the EEA1 staining near the ventricular surface (Figure 1K,L; Figure S1F, Supporting Information). These observations, in conjunction with single‐cell sequencing data, suggest that IFITM2 may play a role in modulating the extensive endocytosis observed in the developing neocortex.

### 2.2 Loss of IFITM2 Promotes Differentiation of RGCs Near the Ventricular Surface

To determine the role of the IFITM2 in developing the neocortex, we engineered a mouse with a conditional *Ifitm2* mutant allele, using the CRISPR‐Cas9 mediated double‐nicking strategy (Figure S2A,B, Supporting Information). These mice were crossed with *Nestin* ^cre^ mice, which express Cre recombinase in neural progenitor cells. Genotyping of pups on the first postnatal day showed a Mendelian ratio, and the pups survived to adulthood. Moreover, *Ifitm2* deletion did not result in high expression compensation in other members of the IFITM family (Figure S2C, Supporting Information).

**Figure 2:**
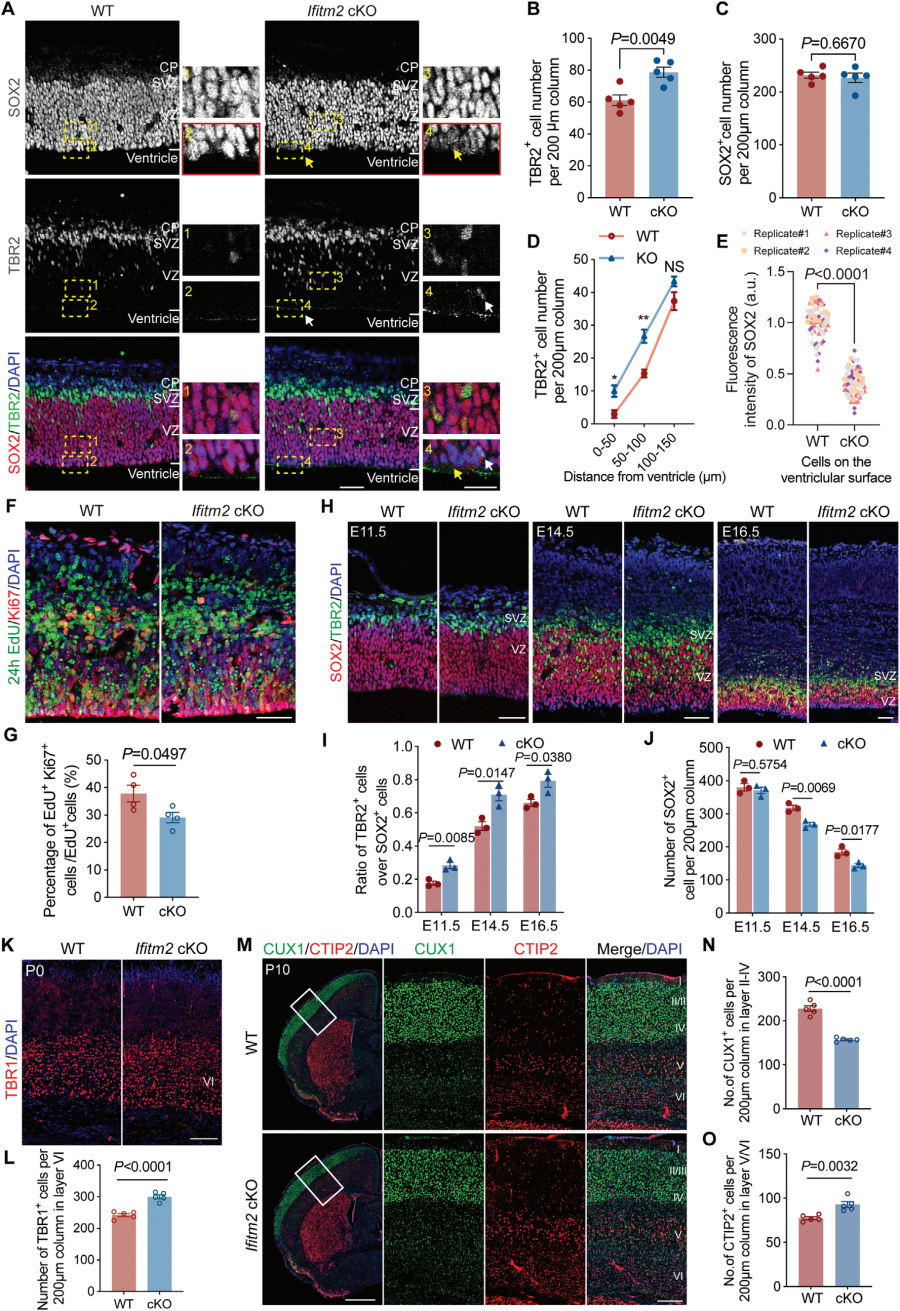
Impact of IFITM2 deletion on neural progenitor dynamics and cortical development. A) Representative images of E12.5 wild‐type and *Ifitm2* cKO cerebral cortex sections stained with SOX2 (red), TBR2 (green), and DAPI (blue). The yellow arrow indicates the weakened signal of SOX2, and the white arrow indicates the appearance of the TBR2 signal near the ventricle area in *Ifitm2* cKO. Scale bars, 50 µm (left) and 20 µm (right). B,C) Quantification of the number of TBR2^+^ cells and SOX2^+^ cells in the wild‐type and *Ifitm2* cKO cortices (*n =* 5 brains per group). Data are shown as mean ± s.e.m. Two‐tailed unpaired Students’ *t*‐test was used.D) Quantification of the number of TBR2^+^ cells in the areas 0–50, 50–100, and 100–150 µm from the ventricle (*n =* 3 brains per group). Data are shown as mean ± s.e.m, ^*^ *p* < 0.05, ^**^ *p* < 0.01. Two‐tailed unpaired Students’ *t*‐test was used. E) Quantification of SOX2 fluorescence intensity in cells located on the surface of the brain ventricle (*n* = 60 cells from 3 brains per group). Two‐tailed unpaired Students’ *t*‐test was used. F) Representative images of wild‐type and *Ifitm2* cKO neocortex that were injected with EdU at E12.5 and analyzed 24 h later. The tissues were stained with EdU (green), Ki67 (red), and DAPI (blue). Scale bar, 50 µm. G) Quantification of the percentage of EdU^+^Ki67^+^ cells among total EdU^+^ cells (*n* = 4 brains per group). Data are shown as mean ± s.e.m. Two‐tailed unpaired Students’ *t*‐test was used. H) Representative images of E11.5, E14.5, and E16.5 wild‐type and *Ifitm2* cKO cerebral cortical sections stained with SOX2 (red), TBR2 (green), and DAPI (blue). Scale bars, 50 µm. I,J) Quantification of the ratio of the TBR2^+^ cells over SOX2^+^ cells (I) and the number of the SOX2^+^ cells (J) (*n =* 3 brains per group). Data are shown as mean ± s.e.m. Two‐tailed unpaired Students’ *t*‐test was used. K) Representative images of P0 wild‐type and *Ifitm2* cKO cerebral cortex sections stained with TBR1 (red), a marker of layer VI neurons, and DAPI (blue). Scale bar, 200 µm. L) Quantification of the number of TBR1^+^ cells in the wild‐type and *Ifitm2* cKO cortices (*n =* 5 brains per group). Data are shown as mean ± s.e.m. Two‐tailed unpaired Students’ *t*‐test was used. M) Representative images of P10 wild‐type and *Ifitm2* cKO brain sections stained with CTIP2 (red), CUX1 (green), and DAPI (blue). White rectangles indicate a dorsal region, which is shown at a higher magnification cortical area in the right. Scale bars, 1 cm (left) and 200 µm (right). N,O) Quantification of the number of CUX1^+^ neurons (N) and CTIP2^+^ neurons (O) per 200 µm column (*n* = 5 brains per group). Data are shown as mean ± s.e.m. Two‐tailed unpaired Students’ *t*‐test was used.

During embryonic neurogenesis, neural stem cells exhibit dynamic nuclear migration, a phenomenon first described by Sauer et al. in 1935.^[^ ^8^ ^]^ This process is characterized by the oscillatory movement of the RGC nucleus during the cell cycle, with mitosis occurring near the ventricular region. To investigate cell fate determination, we focused on the region adjacent to the ventricle, where cells have just completed division (**Figure** **2**A). We found that IFITM2 deficiency led to an increase in TBR2^+^ IPs at E12.5, along with a significant reduction in SOX2 fluorescence near the ventricular surface (Figure 2A–E). Additionally, staining for the neural progenitor cell marker PAX6 showed decreased fluorescence intensity in the same region (Figure S2D,E, Supporting Information). TBR2 expression was found closer to the ventricular surface in the *Ifitm2* conditional knockout (cKO) neocortex compared to wild‐type, further suggesting enhanced differentiation (Figure 2D). These results indicate that the removal of IFITM2 promotes RGC differentiation near the ventricular surface, leading to an increased density of IPs during early neurogenesis.

We then used the FlashTag method that enables in utero labeling of RGCs during mitosis^[^ ^9^ ^]^ to trace the behaviors of RGCs after cell division. The RGC progeny were analyzed 12 h after labeling. FlashTag‐labeled cells in the *Ifitm2* cKO neocortex revealed an increased proportion of TBR2^+^ cells and a decreased presence of SOX2^+^TBR2^−^ cells compared to the wild‐type neocortex (Figure S2F,G, Supporting Information). This result suggests that the removal of IFITM2 leads to an increased differentiation of RGC progeny. Next, we used EdU to label proliferating cells and analyzed them 24 h later. The results showed that IFITM2 deficiency led to a higher proportion of EdU^+^KI67^−^ cells exiting the cell cycle (Figure 2F,G). Additionally, neural progenitor cells isolated from E12.5 cortices revealed that neurospheres formed from *Ifitm2* cKO neural progenitor cells were smaller than those formed from the wild‐type neural progenitor cells, corroborating the in vivo results with similar findings in vitro (Figure S2H,I, Supporting Information).

We subsequently explored whether RGCs are progressively decreased from early to late stages of neurogenesis following the removal of *Ifitm2*. By employing SOX2 and TBR2 immunostaining in the neocortex from E11.5 to E16.5, we observed higher ratios of TBR2^+^ cells over SOX2^+^ cells from early to late neurogenesis (Figure 2H–J), together with fewer SOX2^+^ cells in the E14.5 and E16.5 cerebral cortex of cKO compared with wild‐type mice, indicating a gradual decrease of RGCs. These findings suggest that the removal of IFITM2 leads to increased differentiation of RGCs and reduced RGC pool. Moreover, using EdU labeling to assess neurogenesis in postnatal day (P) 21 and adult mice, we found that the loss of IFITM2 did not significantly affect postnatal neurogenesis (Figure S3A–H, Supporting Information).

During embryonic cortical neurogenesis, the generation of neurons follows a highly orchestrated inside‐out pattern, with deeper layer neurons born first and upper layer neurons born later.^[^ ^10^ ^]^ To examine the impact of increased differentiation of RGCs and reduced RGC pool on the generation of cortical neurons in *Ifitm2* cKO brains, we stained P0 brain sections for TBR1, a marker for layer‐VI pyramidal neurons, and observed an increase in the number of TBR1^+^ deep‐layer neurons, suggesting an increase in early‐born neurons (Figure 2K,L). Then, P10 brain sections were stained for CTIP2, a marker for layer‐V and layer‐VI neurons, and CUX1, a marker for layers II‐IV neurons (Figure 2M–O).^[^ ^11^ ^]^ We found an increased number of CTIP2^+^ neurons, and a significant decreased number of CUX1^+^ neurons in *Ifitm2* cKO brains compared with the wild‐type, indicating an increase in early‐born deep‐layer neurons and a reduction in later‐born superficial‐layer cortical neurons.

### 2.3 Loss of IFITM2 Impairs RGC Endocytosis

**Figure 3:**
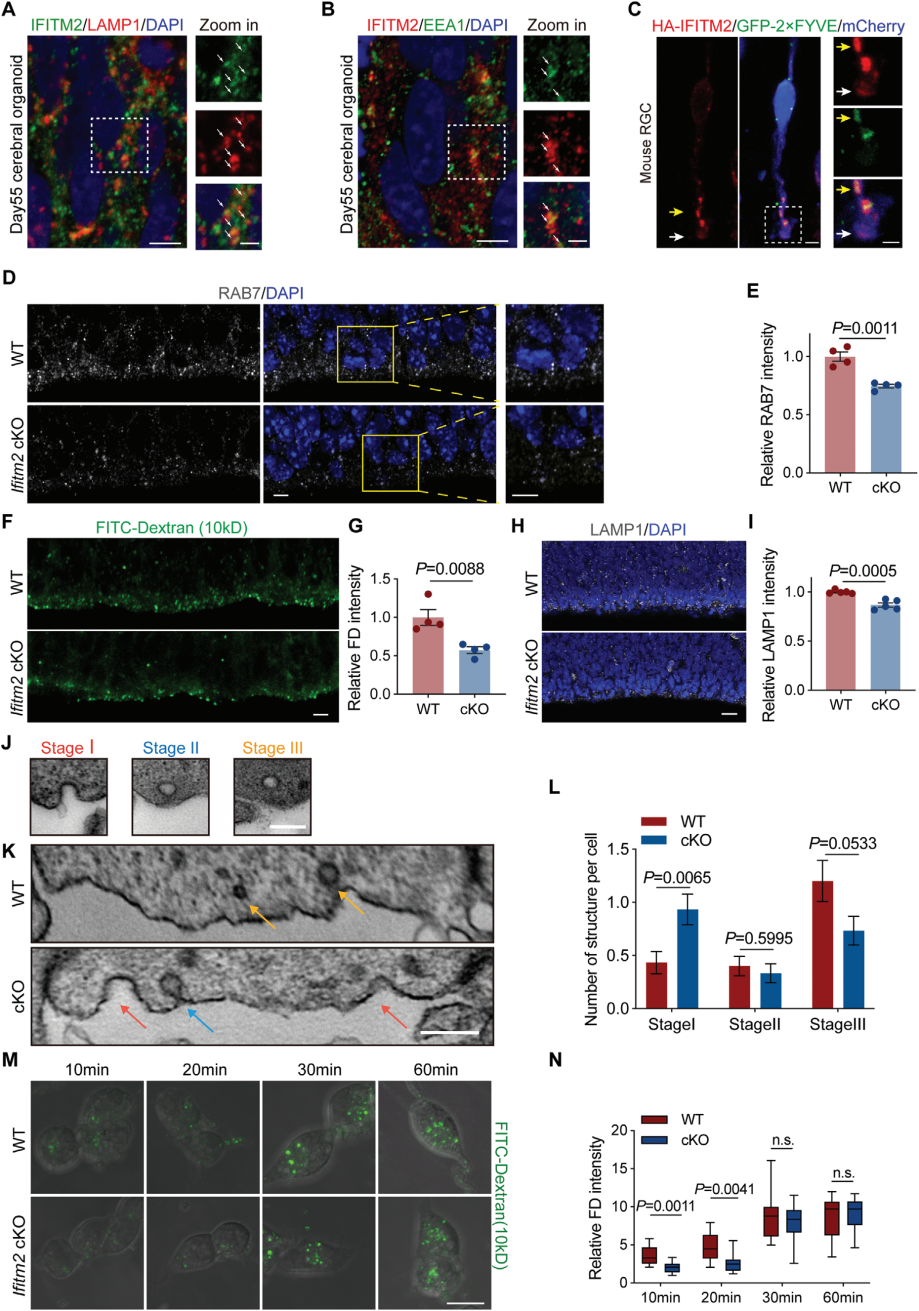
*Ifitm2* deletion disrupts endocytosis on the cortical ventricular surface. A) Representative images of day 55 human cerebral organoid stained with IFITM2 (green), LAMP1 (red), and DAPI (blue). Arrows in the magnified images indicate the colocalization of IFITM2 with LAMP1^+^ lysosomes. Scale bars, 10 and 5 µm. B) Representative images of day 55 human cerebral organoid stained with IFITM2 (red), EEA1 (green), and with DAPI (blue). Arrows in the magnified images indicate the colocalization of IFITM2 with EEA1^+^ endosomes. Scale bars, 10 and 5 µm. C) Representative images of RGC introduced with HA‐IFITM2 (red), GFP‐2 × FYVE (green), and mCherry (blue) in mice. The white arrow in magnified image indicates the apical polarized localization of IFITM2 in RGC, while the yellow arrow indicates the colocalization of IFITM2 with PI3P^+^ vesicle. Scale bars, 5 and 2 µm. D) Representative images of the E14.5 wild‐type and *Ifitm2* cKO stained with RAB7 (gray) in the ventricular zone. Scale bars, 50 µm. E) Quantification of relative fluorescence intensity for RAB7 (*n* = 4 brains per group). Data are shown as mean ± s.e.m. Two‐tailed unpaired Students’ *t*‐test was used. F) Representative images of E14.5 wild‐type and *Ifitm2* cKO brains 20 min after injecting 10 kD FITC‐Dextran. Scale bar, 10 µm. G) Quantification of the FITC‐Dextran fluorescence intensity (*n* = 4 brains per group). Data are shown as mean ± s.e.m. Two‐tailed unpaired Students’ *t*‐test was used. H) Representative images of E14.5 the wild‐type and *Ifitm2* cKO stained with LAMP1, a marker for endolysosome. Scale bar, 20 µm. I) Quantification of relative fluorescence intensity of LAMP1 in the VZ region (*n* = 5 brains per group). Data are shown as mean ± s.e.m. Two‐tailed unpaired Students’ *t*‐test was used. J) Representative TEM images of three stages of the endocytosis process on the cortical ventricular surface. Scale bar, 100 nm. K) Representative TEM images of the E14.5 wild‐type and *Ifitm2* cKO cortical ventricular surface. The red arrows indicate U‐shaped stage one, the blue arrow indicates the Ω‐shaped stage two, and the yellow arrows indicate the endo‐vesicles of stage three. Scale bar, 250 nm. L) Quantification the number of structures in three stages in each cell (*n* = 30 cells from 3 embryos per group). Data are shown as mean ± s.e.m. Two‐tailed unpaired Students’ *t*‐test was used. M) Representative images of wild‐type and *Ifitm2* cKO primary neural stem cells endocytosing 10 kD FITC‐Dextran. The samples were collected at 10, 20, 30, and 60 min. Scale bar, 10 µm. N) Quantification of the relative FITC intensity (*n* = 12 cells per group). Data are shown as mean ± s.e.m. Two‐tailed unpaired Students’ *t*‐test was used.

In human‐derived cerebral organoids, we observed the co‐localization of IFITM2 with both EEA1 and LAMP1, confirming its localization to early endosomes and endolysosomes (**Figure** **3**A,B). Additionally, by electroporating HA‐IFITM2 and GFP‐2 × FYVE into mouse RGCs, which labels phosphatidylinositol 3‐phosphate (PI3P)‐containing endocytic vesicles, we observed that IFITM2 co‐localized with PI3P^+^ vesicles in RGCs, with the distribution of IFITM2 also observed near the ventricular surface (Figure 3C). To investigate the effects of *Ifitm2* deletion on the endocytic process, we performed immunostaining for RAB7, a late endosome marker, and found a significant reduction in the number of late endosomes in the *Ifitm2* cKO cortices (Figure 3D,E). To further confirm the impact on endocytosis, we injected 10 kD FITC‐dextran into the lateral ventricle of E14.5 brains and collected samples after 20 min. A marked reduction in fluorescence intensity of FITC‐dextran was observed in the *Ifitm2* cKO neocortex compared to the wild‐type, indicating a reduced uptake of dextran (Figure 3F,G). Our previous work demonstrated that endolysosomes are the predominant type of lysosomes in embryonic RGCs.^[^ ^2^ ^]^ Here, using LAMP1 to label lysosomes, we found that IFITM2 deletion led to a significant reduction in the number of lysosomes (Figure 3H,I). Together, these findings underscore the crucial role of IFITM2 in regulating endocytosis during early cortical neurogenesis.

We next performed transmission electron microscopy (TEM) to visualize the membrane structure on the cortical ventricular surface (Figure S4, Supporting Information). Endocytosis exhibits three stages: stage one involves the formation of a U‐shaped invagination in the cell membrane, stage two entails the contraction of the cell membrane into an Ω‐shaped half‐vesicle, and stage three represents the completion of membrane contraction during endocytosis (Figure 3J).^[^ ^12^ ^]^ Comparing the wild‐type and the *Ifitm2* cKO neocortices, we found that the knockout of IFITM2 resulted in an increase in stage one, a slight but insignificant reduction in stage two, and a significant reduction in stage three (Figure 3K,L). This result suggests that deletion of IFITM2 delays the transition from stage one to stage two, that is, the closure of endocytosis vesicles. Furthermore, in vitro experiments conducted on primary NPCs revealed a decrease in endocytosis of the dextran in *Ifitm2* cKO cells compared with wild‐type cells at 10 and 20 min, while there was no difference after 30 min, suggesting that IFITM2 is a rate‐limiting factor for endocytosis (Figure 3M,N). Together, these results suggest that IFITM2 deletion hinders the endocytosis near the brain ventricle surface, specifically delaying endocytic vesicle closure.

### 2.4 Structural Insights and Functional Regulation of IFITM2 in Endocytosis

**Figure 4:**
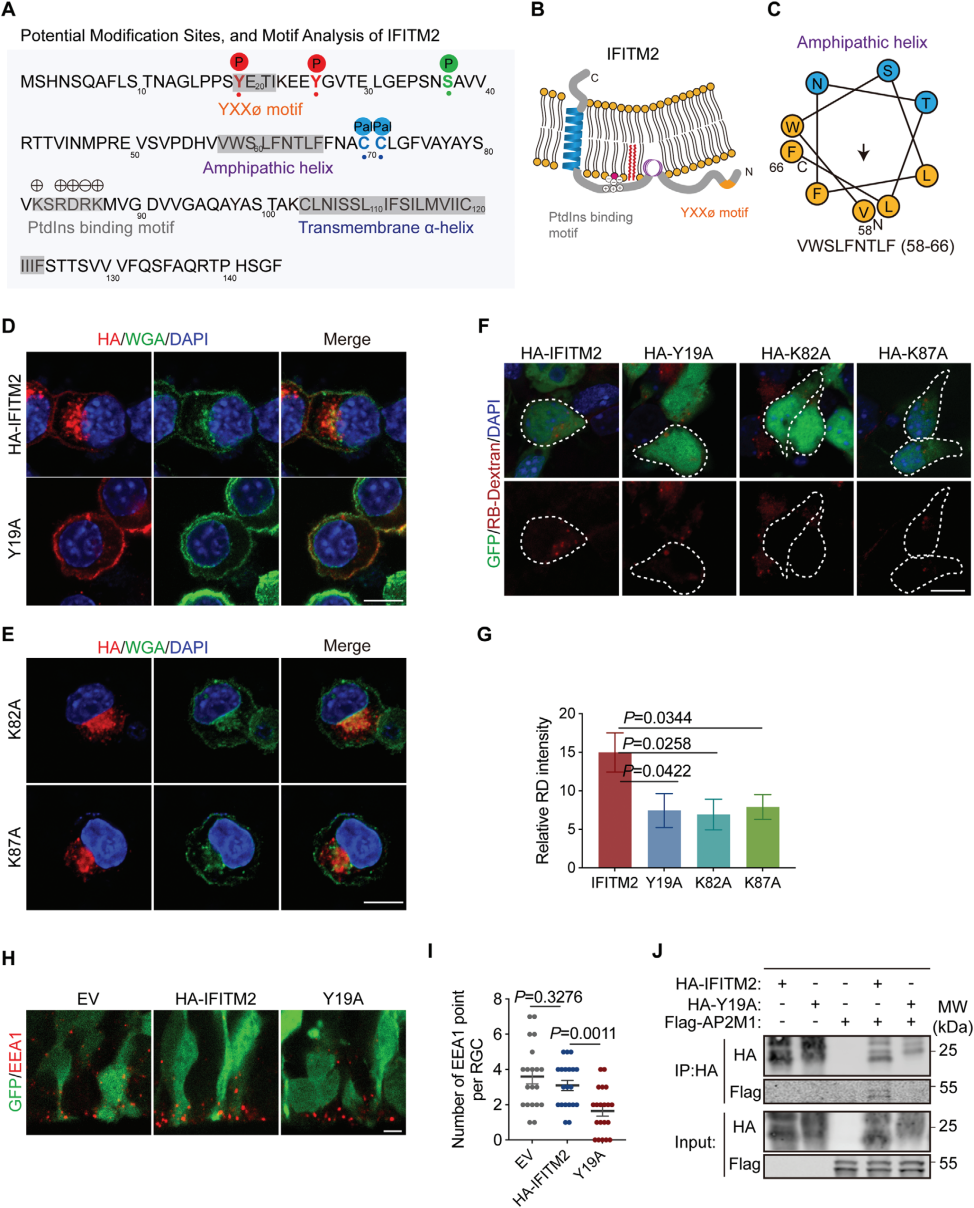
Structural features and functional mechanisms of IFITM2 in endocytosis regulation. A) Amino acids sequence and potential modification sites of mouse IFITM2 protein. B) Predicted binding model of IFITM2 to the plasma membrane. C) Helical wheel projection plots of the amphipathic helix of IFITM2 protein. Hydrophobic residues are displayed as yellow, and polar uncharged residues are displayed as blue. Arrow represents the magnitude and orientation of the mean hydrophobic moment. D,E) Representative images of Neuro‐2a cells transfected with HA‐IFITM2 (D), HA‐Y19A mutant (D), HA‐K82A mutant (E), or HA‐K87A mutant (E), and stained with HA antibody (red), Wheat Germ Agglutinin (green), and DAPI (blue). Scale bars, 10 µm. F) Representative images of primary neural progenitor cells transfected with HA‐IFITM2, HA‐Y19A, HA‐K82A, or HA‐K87A lentivirus for 2 days, followed by incubation with 10 kD RB‐Dextran for 20 min. Scale bar, 10 µm. G) Quantification of the fluorescence intensity of endocytic RB‐Dextran signals per cell (*n* = 8 cells per group). Data are shown as mean ± s.e.m. Two‐tailed unpaired Students' *t*‐test was used. H) Representative images of RGCs electroporated with the GFP reporter (green) and EV, HA‐IFITM2, or HA‐Y19A. Sections were stained with EEA1 (red). Scale bar, 10 µm. I) Quantification of the number of EEA1 in RGC (*n* = 20 cells from 3 embryos per group). Data are shown as mean ± s.e.m. Two‐tailed unpaired Students' *t*‐test was used. J) Co‐immunoprecipitation assays were performed to detect the interaction between AP2M1 and IFITM2 or Y19A using HA‐tagged beads in HEK293T cells.

The functionality of a protein is intrinsically linked to its structural configuration. Through a detailed analysis of the IFITM2 amino acid sequence, we identified several key structural features, including a single transmembrane domain, an amphipathic helix, a YXXø endocytic motif, and a phosphoinositide (PtdIns) binding motif (**Figure** **4**A). Structural modeling of IFITM2 further suggests that it is a single‐pass transmembrane protein, with its N‐terminal region predominantly localized intracellularly, while the C‐terminal extracellular domain is composed of only a few amino acids, and thus this topology indicates that IFITM2 is unlikely to interact with extracellular ligands (Figure 4B).

The amphipathic helix is characterized by the juxtaposition of hydrophilic and hydrophobic residues on opposite faces of the helix, enabling it to interact with both aqueous environments and lipid membranes (Figure 4C).^[^ ^4d^ ^]^ This amphipathic nature facilitates the formation of a “wedge” effect, wherein the helix inserts into one leaflet of the bilayer, thereby inducing changes in membrane curvature—a crucial mechanism in vesicle formation.

In addition, the YXXø endocytic motif and the PtdIns binding motif appear to be pivotal regulatory elements governing the function of IFITM2. We initially focused on investigating the YXXø endocytic motif. Through mutation screening of potential phosphorylation sites, we found that the Y19A mutant predominantly localized at the plasma membrane, and thus pinpointed the Y19 residue within the YXXø motif as a critical phosphorylation site (Figure 4D; Figure S5A,B, Supporting Information). Electroporation of the Y19A mutant into cells, followed by dextran uptake assays, revealed that overexpression of Y19A significantly impaired endocytosis, indicating that this mutation disrupts IFITM2's functional capacity (Figure 4F,G). In addition, in vivo analysis demonstrated a substantial reduction in the number of endocytic vesicles in RGCs expressing the Y19A mutant (Figure 4H,I). Importantly, the phosphorylation of the Y19 residue is consistent with the conserved YXXø motif, which is a hallmark of the IFITM family.^[^ ^13^ ^]^ This motif serves as a sorting signal in the cytosolic tails of transmembrane proteins, orchestrating the recruitment of adaptor protein complexes essential for clathrin‐coated vesicle formation at the plasma membrane. The Y19A mutation, however, abolishes the interaction with adaptor protein 2 (AP2), thereby inhibiting vesicle formation (Figure 4J). Together, these findings suggest that the YXXø motif of IFITM2 is a critical phosphorylation site that regulates endocytosis in neural progenitor cells.

Additionally, previous studies have demonstrated that IFITM3 acts as a scaffold for phosphoinositides, amplifying their signaling.^[^ ^14^ ^]^ Given the high sequence conservation between IFITM2 and IFITM3, we hypothesized that IFITM2 might also play a similar role. We investigated the PtdIns binding motif by introducing mutations at K82 and K87, which led to the loss of IFITM2 localization at the plasma membrane (Figure 4E). We introduced these mutants alongside GFP‐2 × FYVE into the developing neocortex and observed that the mutations significantly reduced co‐localization with GFP‐2 × FYVE^+^ vesicles and diminished their size (Figure S5C–E, Supporting Information). These results underline the critical roles of residues K82 and K87 in binding PtdIns and regulating endocytosis. Together, these findings suggest that the PtdIns binding motif of IFITM2 is also critical for its regulation of endocytosis. Encouraged by these results, it is necessary to further understand the roles of phosphoinositides in the developing neocortex.

### 2.5 Regulation of Endocytosis by Phosphoinositides and IFITM2 in Developing Neocortex

**Figure 5:**
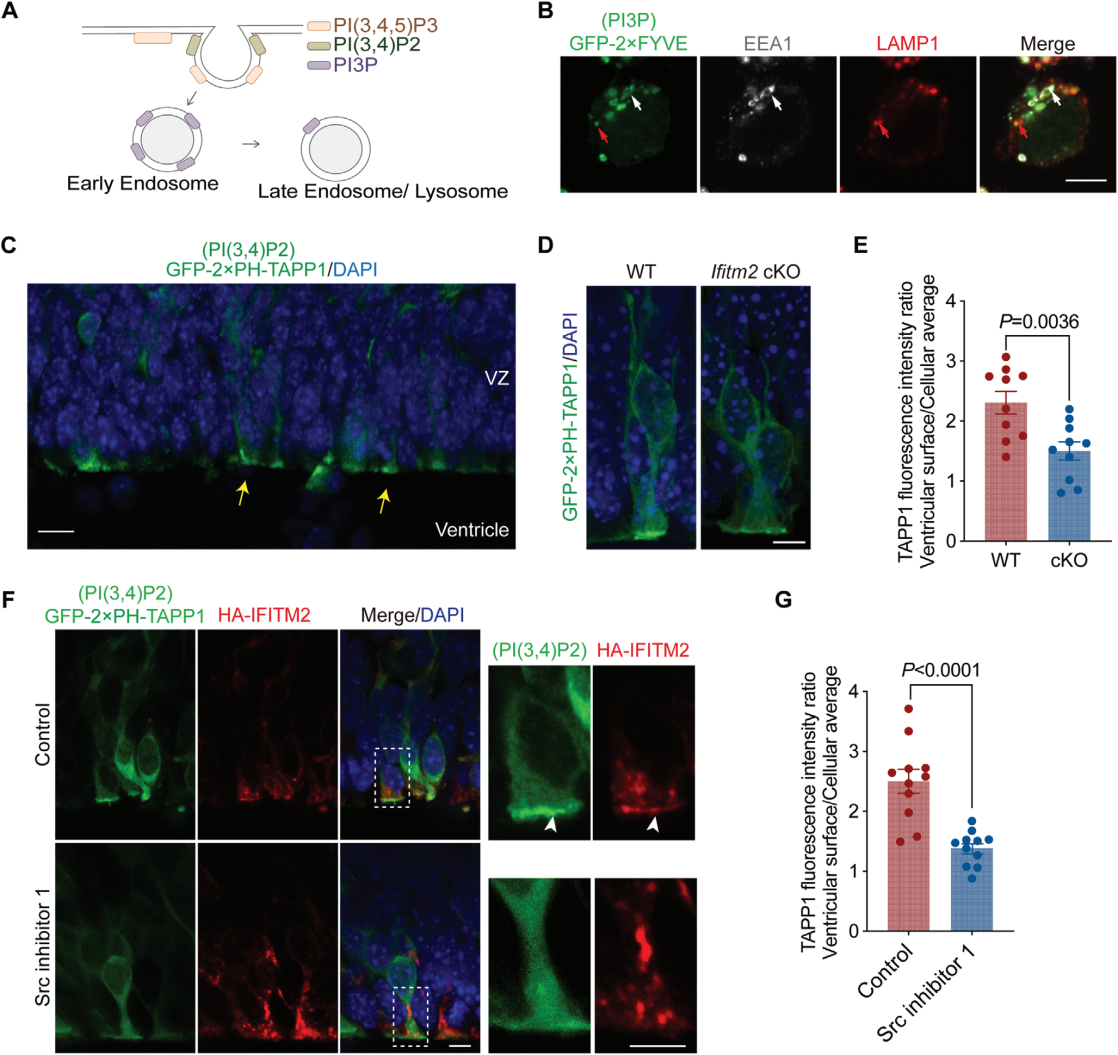
IFITM2 modulates PI(3,4)P2 signaling to regulate endocytosis in RGCs. A) Schematic diagram of the relation between the endocytosis process and different types of phosphoinositide. B) Representative images of Neuro‐2a cells transfected with GFP‐2 × FYVE (green) and stained with EEA1 (white) and LAMP1 (red). White arrows indicate the colocalization of GFP‐2 × FYVE and EEA1^+^ early endosome, and red arrows indicates the co‐localization of GFP‐2 × FYVE and LAMP1^+^ lysosome. Scale bar, 5 µm. C) Representative IUE image of neocortex ventricular zone electroporated with the GFP‐2 × PH‐TAPP1 (green) at E13.5, analyzed at E15.5. Yellow arrows indicate an enrichment of PI(3,4)P2 signaling at the ventricular surface. Scale bar, 10 µm. D) Representative IUE images of the E14.5 wild‐type and *Ifitm2* cKO neocortex electroporated with GFP‐2 × PH‐TAPP1 (green) and HA‐IFITM2 (red) plasmids. Scale bar, 5 µm. E) Quantification of the ratio of fluorescence intensity of TAPP1 at the ventricular surface to the average fluorescence intensity across the entire cell (*n* = 10 cells per group). Data are shown as mean ± s.e.m. Two‐tailed unpaired Students’ *t*‐test was used. F) Representative IUE images of the neocortex electroporated with GFP‐2 × PH‐TAPP1 (green) and HA‐IFITM2 (red) plasmids. A 10 µm concentration of Src inhibitor 1, an ATP‐competitive and selective dual‐site Src tyrosine kinase inhibitor, was administered into the lateral ventricle 20 min prior to tissue collection. Arrow heads indicate the co‐localization of PI(3,4)P2 signaling with HA‐IFITM2 at the apical membrane of RGCs. Scale bars, 5 µm. G) Quantification of the ratio of fluorescence intensity of TAPP1 at the ventricular surface to the average fluorescence intensity across the entire cell after Src inhibitor 1 treatment (*n* = 11 cells per group). Data are shown as mean ± s.e.m. Two‐tailed unpaired Students’ *t*‐test was used.

Phosphoinositide signaling plays a pivotal role in the regulation of endocytosis by modulating vesicle formation and trafficking (**Figure** **5**A). PI3P primarily localizes to intracellular vacuoles, while PI(3,4)P2 and phosphatidylinositol trisphosphate (PI(3,4,5)P3) predominantly localize to the plasma membrane.^[^ ^15^ ^]^ In particular, PI(3,4)P2 is crucial for membrane constriction and the formation of endocytic vesicles.^[^ ^16^ ^]^

We first confirmed that PI3P co‐localized with EEA1^+^ early endosomes and LAMP1^+^ endolysosomes in Neuro‐2a cells (Figure 5B). Building on these observations, we then explored the role of PI(3,4)P2 in the developing neocortex. By introducing GFP‐2 × PH‐TAPP1, a specific probe for PI(3,4)P2, into RGCs via in utero electroporation, we observed a polarized distribution of PI(3,4)P2 signaling at the ventricular surface (Figure 5C). Next, we investigated the potential role of IFITM2 as a scaffold for phosphoinositides and its impact on this polarized distribution of PI(3,4)P2 signaling. In Ifitm2 cKO brains, we found that the absence of IFITM2 resulted in a significant reduction in PI(3,4)P2 signaling on the apical side of RGCs (Figure 5D,E). This finding suggested that IFITM2 functions as a PI(3,4)P2 scaffold, which is crucial for membrane constriction and the formation of endocytic vesicles.

We next explored the relationship between IFITM2 phosphorylation and PI(3,4)P2 signaling. Src kinase, a known activator of IFITM2,^[^ ^17^ ^]^ was inhibited in the developing cerebral cortex using Src inhibitor 1. This treatment resulted in the loss of IFITM2's polarized localization and a reduction in PI(3,4)P2 signaling at the ventricular surface (Figure 5F,G). These findings collectively suggest that Src kinase promotes the polarization of IFITM2, which in turn stabilizes PI(3,4)P2 signaling, highlighting the complex interaction between IFITM2 and phosphoinositide signaling in regulating endocytic processes.

### 2.6 IFITM2 Modulates FAK‐PI3K‐AKT Signaling in Neural Development

**Figure 6:**
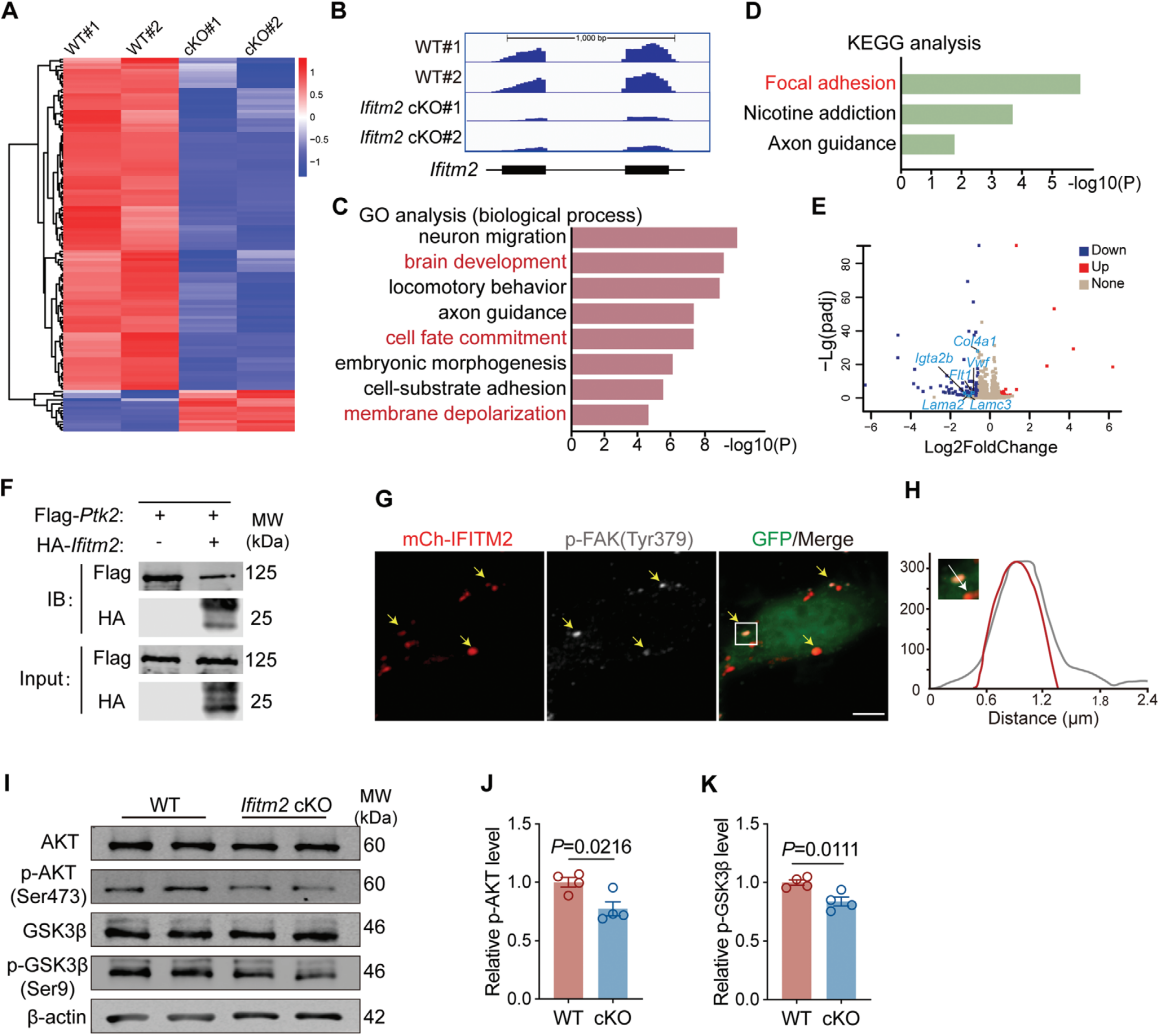
IFITM2 regulates focal adhesion and PI3K‐AKT signaling in the developing neocortex. A) Heat map of the differentially expressed transcripts in wild‐type and *Ifitm2* cKO E13.5 neocortex. The genes are clustered according to the degree of expression similarity on the left. The red and blue colors indicate upregulation and downregulation, respectively. B) *Ifitm2* transcript expression of wild‐type and *Ifitm2* cKO by RNA sequencing. C) Gene ontology (GO) enrichment analysis of the down‐regulated genes for biological process (BP). D) Kyoto Encyclopedia of Genes and Genomes (KEGG) pathway enrichment analyses of down‐regulated genes. E) Volcano plots illustrating the downregulated (blue) and up‐regulated (red) genes in the *Ifitm2* cKO brain neocortex compared with the wild‐type cortex. Representative differentially expressed genes associated with the FAK pathway are labeled. F) The co‐immunoprecipitation experiments to detect the interaction between IFITM2 and PTK2 with Flag‐tag beads in HEK293T cells. G) Representative images of the neural progenitor cells electroporated with mCherry‐IFITM2 (red) and GFP (green) and stained with p‐FAK (Tyr379) (gray). Yellow arrows indicate the colocalization of mCh‐IFITM2 and p‐FAK (Tyr379). Scale bar, 5 µm. H) Intensities of p‐FAK (Tyr379) (gray) and mCh‐IFITM2 (red) on the arrow. I) Western blot analysis of AKT, p‐AKT, GSK3β, and p‐GSK3β levels in E13.5 cortices from wild‐type and *Ifitm2* cKO mice. J,K) Qualification of the p‐AKT and p‐GSK3β level (*n* = 4 independent experiments). Data are shown as mean ± s.e.m. Two‐tailed unpaired Students’ *t*‐test.

Finally, we explored which signaling pathways are affected at the transcriptional level in IFITM2‐mediated endocytosis. To investigate this, we performed RNA sequencing (RNA‐seq) to compare differentially expressed transcripts between the E13.5 wild‐type and *Ifitm2* cKO neocortex (**Figure** **6**A). The knockout efficiency of *Ifitm2* was validated (Figure 6B). We found a smaller number of upregulated genes, and neither KEGG nor GO analysis revealed specific pathways for these genes. In contrast, GO analysis of the downregulated genes showed a strong association between IFITM2 and brain development, cell fate transitions, and cell membrane polarity (Figure 6C). Furthermore, KEGG pathway enrichment analysis identified significant changes in the focal adhesion pathway (Figure 6D). Representative genes related to focal adhesion were shown in the volcano plots (Figure 6E). The focal adhesion pathway is crucial for regulating cell proliferation, adhesion, spreading, and survival.^[^ ^18^ ^]^ In this context, phosphorylation of focal adhesion kinase (phospho‐FAK) triggers the phosphoinositide 3‐kinase (PI3K)‐AKT signaling cascade, which is essential for cell growth and proliferation.^[^ ^19^ ^]^ Additionally, introducing a knockdown vector for FAK, *Ptk2*‐shRNA, into mouse cortices via in utero electroporation promoted neural differentiation (Figure S6A–C, Supporting Information).

Then, we performed co‐immunoprecipitation assays to explore the interaction between IFITM2 and FAK in 293T cells. Our results confirmed that Flag‐tagged FAK robustly pulled down HA‐tagged IFITM2 (Figure 6F). Immunofluorescence staining further demonstrated that phospho‐FAK (Tyr379) co‐localized with IFITM2 in primary neural progenitor cells (Figure 6G,H). To elucidate the role of IFITM2 in the FAK‐PI3K‐AKT signaling pathway, we performed immunoblotting analysis. The results revealed decreased phosphorylation levels of AKT and GSK3β in *Ifitm2* cKO neocortex compared to the wild‐type (Figure 6I–K). It is well‐established that endocytosis faciliates the activation of AKT on endosomes, which in turn inhibits GSK3β activity.^[^ ^20^ ^]^ Thus, the reduced endocytic activity leads to decreased AKT activation and increased activation of GSK3β in *Ifitm2* cKO neocortex, affecting cellular processes such as proliferation and growth. In addition, despite these signaling changes, we observed no significant alterations in the actin cytoskeleton within the ventricular zone or at the ventricular surface, suggesting that the loss of IFITM2 predominantly affects endocytosis and associated signaling without markedly influencing actin dynamics (Figure S6D–H, Supporting Information).

## 3 Discussion

During early embryonic neural development, endocytosis plays a crucial role in the internalization of key signaling molecules and receptors for neural stem cells. This study provides substantial evidence supporting the idea that endocytosis is vital for maintaining stemness in neural stem cells. Moreover, we have demonstrated a non‐immune role of IFITM2 in regulating endocytosis in the mouse and human developing neocortex.

Endocytosis in the developing cortex regulates receptor internalization, degradation, and responses to extracellular environments.^[^ ^21^ ^]^ For example, overexpression of Numb promotes trafficking and degradation of Notch 1.^[^ ^22^ ^]^ Furthermore, Numb mutants with defective binding to endocytic proteins like α‐adaptin fail to promote Notch 1 degradation. However, we still have a limited understanding of the regulatory mechanisms that initiate endocytosis in RGCs. Here we offered a mechanistic insight, showing that IFITM2 enhances endocytosis through its interaction with PI(3,4)P2 signaling activated by FAK. Consequently, IFITM2 deficiency inhibited endocytosis in RGCs and promoted their differentiation.

Recent studies have extensively investigated the IFITM family, focusing on their roles in immunology and oncology. In terms of immunity, IFITMs are known for their antiviral function, which is reported to inhibit viral entry by inducing negative membrane curvature.^[^ ^23^ ^]^ In cancer, IFITMs are identified as predictors of poor prognosis in several types, including melanoma, glioma, leukemia, lymphoma, and pancreatic ductal adenocarcinoma.^[^ ^24^ ^]^ IFITMs have also recently emerged as markers of therapeutically resistant and aggressive cancers.^[^ ^14^, ^25^ ^]^ Interestingly, interferons can induce the expression of IFITMs during combating viral infections and can also activate anti‐cancer immune responses in immunotherapy.^[^ ^26^ ^]^ Considering the role of IFITM2 in regulating endocytosis in the developing neocortex, it is possible that IFITMs also affect endocytosis in cancer cells, potentially leading to abnormal cell proliferation and the loss of cell surface antigens. Thus, in clinical settings, combining interferon therapy with inhibition of IFITM expression may yield better outcomes.

Mechanistically, the topology of IFITM proteins suggests that they are unlikely to have natural ligands. Three different affinity proteomic studies found different sets of IFITM interacting proteins with minimal overlap.^[^ ^27^ ^]^ Consequently, we hypothesized that IFITM2 might primarily interact with lipid signaling pathways. This is supported by a study in B cell leukemia and lymphoma, which found that IFITM3 could act as a PIP3 scaffold.^[^ ^14^ ^]^ In our research, we discovered that IFITM2 binds to PI(3,4)P2 on the mouse embryonic brain ventricular surface. This interaction involves the K82 and K87 basic residues of IFITM2, which are proximal to two potential palmitoylation modification sites and an amphipathic helix. We speculated that the phosphatidylinositol signals may regulate the reversible post‐translational modifications of IFITM2, subsequently promoting the insertion of the amphipathic helix into the lipid bilayer, ultimately promoting membrane curvature (Figure S7A, Supporting Information). This mechanism might explain that IFITM2 removal led to a TEM‐observed increase in type I endocytic vesicles and a decrease in type III endocytic vesicles on the ventricular surface.

Despite the insights from this study, several questions remain unanswered. For instance, how the specific extracellular matrix proteins and integrins interact to regulate endocytosis on the brain ventricle surface is still unclear. Through digging RNA in situ hybridization database, we found high expression of ECM proteins, such as *Vtn*, at the ventricular surface, and integrins like *Itgav* and *Itga6* at the ventricular zone (Figure S7B–D, Supporting Information).^[^ ^28^ ^]^ The regulatory mechanisms of these region‐specific ECM and integrin proteins warrant further systematic investigation. Furthermore, the role of endocytosis in neurogenesis is a vast area of study, with previous research on molecules like Numb and Notch also implicating endocytosis. More recent studies suggest that the subcommissural organ regulates brain development via secreted peptides, with gene knockout of these peptides causing significant cortical developmental defects.^[^ ^29^ ^]^ The interactions between RGCs, the cerebrospinal fluid, and the extracellular matrix are key areas requiring more in‐depth exploration. Additionally, while our study focused on endocytosis at the ventricular surface, the potential role of IFITM2 in intracellular vesicle trafficking remains unexplored. Given that the anti‐viral strategies of IFITMs are involved in inhibiting endosome‐lysosome fusion,^[^ ^13a^ ^]^ the potential role of IFITM2 in vesicular transport cannot be ruled out. Lastly, the molecular mechanisms underlying IFITM2's functions remain unclear; however, new techniques such as DNA PAINT,^[^ ^30^ ^]^ cryo‐electron tomography (ET),^[^ ^31^ ^]^ and cryo‐correlative electron and light microscopy (CLEM)^[^ ^32^ ^]^ may provide high‐resolution in situ structural insights, further advancing our understanding of the structural biology of IFITMs in membrane dynamics.

## 4 Experimental Section

### Mouse

The *Ifitm2* conditional knockout mice were generated by using the CRISPR‐CAS9 mediated double nicking strategy. Guide RNA sequence: gRNA1 (5′‐ATGAAACTGCCTGGGGATTCTGG‐3′); gRNA2 (5′‐ AGGCTTCTGGTCTCCAGATATGG‐3′). Nestin^cre^ mice were used to recognize and cut the flox site. All animal experiments were approved by the Animal Care and Use Committees of the Institute of Zoology, Chinese Academy of Sciences. The ethical approval number for animal studies is IOZ20190080.

### Plasmids

Mouse IFITM2 cDNA (accession number NM_030694) was obtained by polymerase chain reaction (PCR) and cloned into pCAG‐HA to generate pCAG‐HA‐IFITM2. Mouse LAMP2 cDNA (accession number NM_001017959) and RAB7A cDNA (accession number AB232599) were obtained by polymerase chain reaction (PCR) and cloned into pCAG‐N1‐mCherry/GFP to generate pCAG‐LAMP2‐mCherry. Y19A plasmid was obtained by homologous recombinant HA‐IFITM2. Mouse GRP1 cDNA (accession number NM_001163548) was obtained by polymerase chain reaction (PCR) and cloned into pCAG‐GFP to generate pCAG‐GFP‐2 × FYVE and pCAG‐GFP‐2 × PH‐ TAPP1 were cloned from pEGFP‐2xFYVE (MIAOLING Plasmid, #P31053) and GFP‐2 × PH‐TAPP1 (MIAOLING Plasmid, #P36199). The shRNA fragments were synthesized and constructed into pSicoR. The sequences of shRNAs targeting PTK2 are as follow: *Ptk2*‐sh1, 5′‐GCCTTAACAATGCGTCAGTTT‐3′, and *Ptk2*‐sh2, 5′‐CCTGGCATCTTTGATATTATA‐3′.

### Immunostaining

Brains were fixed with 4% PFA (4% paraformaldehyde dissolved in PBS) overnight and then transferred into 30% sucrose to dehydrate. Brains were sectioned at 15 µm for immunostaining. For immunostaining, use 4% PFA for 20 min at room temperature, and wash 3 times with PBST (10% Triton X‐100) for 10 min. Decant 5% BSA in PBST for 2 h at room temperature, then change with the primary antibodies overnight. Wash the slices 3 times for 10 min and incubate them with secondary antibodies for 2 h at room temperature in the dark. In the end, wash the slices in PBS and seal them with 50% glycerin. For some immunostaining, nuclei were labeled by DAPI (Sigma, Cat# D9542) for 2 min before the final seal. For EdU staining, the BeyoClick EdU Cell Proliferation Kit with Alexa Fluor 488 (Beyotime Cat#C0071S) was used following the manufacturer's instructions.

### Human Cerebral Organoid Generation

The STEM diff cerebral organoid kits (STEM, Cat#08570, Cat#08571) were used to generate cerebral organoids. All steps were followed by the protocol of the products. There are four steps: embryoid body formation, induction, Expansion, and organoid maturation. The H9 ES cell line was used to form EBs. In the first three steps, the EBs were cultured in the 96‐well or 24‐well low attachment plate. On day 12, the tissues were transferred into a 3D spinning bioreactor (Pfeiffer spinner, Cat# 183001).

### FlashTag Labeling

FlashTag labeling was performed as previously described.^[^ ^2^ ^]^ Briefly, timed pregnant mice were anaesthetized with 2,2,2‐tribromoethanol and 2‐Methyl‐2‐butanol, and the uterine horns were exposed for the experiment. A 2 mm solution of carboxyfluorescein succinimidylester (CellTrace, Thermo Fisher, Cat# C34570) was injected into the lateral ventricle of the embryonic brain to label the M‐phase apical juxtaventricular radial glial cells. The CellTrace compounds function by covalently binding the intracellular proteins with the fluorescent dye carboxyfluorescein. The term FT refers to both these compounds and the pulse‐labeling procedure.

### Antibodies

Rabbit anti‐SOX2 (Cell Signaling Technology Cat# 3728S; RRID: AB_2194037); Rabbit anti‐IFITM2 (Proteintech Cat# 12769‐1‐AP; RRID: AB_2122089); Mouse anti‐IFITM2 (Santa Cruz Biotechnology Cat# sc‐373676; RRID: AB_10915739); Rabbit anti‐TBR1 (Abcam Cat# ab31940; RRID: AB_2200219); Rat anti‐CTIP2 (Abcam Cat# ab18465; RRID: AB_2064130); Rabbit anti‐CUX1 (Proteintech Cat# 11733‐1‐AP; RRID: AB_ 2086995); Mouse anti‐TUJ1 (Millipore Cat# MAB1637; RRID: AB_ 2210524); Rabbit anti‐PAX6 (Millipore Cat# AB2237; RRID: AB_ 1587367); Rat anti‐TBR2/EOMES (eBioscience Cat# 14‐4875‐82; RRID: AB_11042577); Rabbit anti‐Ki67 (Abcam Cat# ab15580; RRID: AB_443209); Rabbit anti‐Rab7 (Cell Signaling Technology Cat# 9367; RRID: AB_1904103); Rat anti‐LAMP1 (BD Biosciences Cat# 553792; RRID: AB_2134499); Rabbit anti‐EEA1 (Cell Signaling Technology Cat# 3288T; RRID: AB_2096811); Rabbit anti‐HA (Cell Signaling Technology Cat# 3724S; RRID: AB_1549585); Rabbit anti‐FAK (ABclonal Cat# A11195; RRID: 2758455); Rabbit anti‐p‐FAK(Y397) (ABclonal Cat# AP0302; RRID: 2771470); Rabbit anti‐p‐GSK‐3β (S9) (Cell Signaling Technology Cat# 5558T; RRID: AB_10013750); Rabbit anti‐AKT (Cell Signaling Technology Cat# 4865S; RRID: 2225340); Rabbit anti‐p‐AKT(Ser473) (Cell Signaling Technology Cat# 3787S; RRID: 331170); Rabbit anti‐β‐actin (Proteintech Cat#20536‐1‐AP; RRID: AB_10700003); PE/Cyanine7 anti‐mouse CD133 Antibody (BioLegend Cat# 141209; RRID: AB_2564068); FITC anti‐mouse CD24 Antibody (BioLegend Cat# 101806; RRID: AB_312839); 594‐Phalloidin (Proteintech Cat#PF00003; RRID: AB_3675292); Wheat Germ Agglutinin (Thermo fisher scientific Cat# W11261).

### Transmission Electron Microscopy (TEM)

TEM was performed as previously described.^[^ ^33^ ^]^ The E15.5 wild‐type and *Ifitm2* cKO cortices were cut into sections and fixed with 2% PFA and 2.5% glutaraldehyde overnight at 4 °C. After washing with PBS three times, the sections were post‐fixed in 1% osmium for 1 h. Then washed and dehydrated by an ethanol series. Use 100% acetone to wash sections, followed by soaking in acetone and epoxy resin. The sections were embedded with epoxy resin for 48 h. Serial sections of 60 nm were cut by Leica UC7 ultramicrotome. Images of the brain ventricular surface were acquired with a transmission electron microscope (Tecnai G2 F20 TWIN TMP).

### Cells

The Neuro‐2a cells and HEK 293T cells were cultured in high glucose DMEM (Gibco) with 1% penicillin/ streptomycin (Invitrogen) and 10% fetal bovine serum (Gibco). The H9 cells (hECs) were cultured in the Essential 8 medium kit (Thermo). The primary neural progenitor cells were isolated from the E12.5 cortices.

### Flow Cytometry

Neural stem cells were isolated from embryonic mouse cortices in a sterile bench. The tissue was first dissociated using a papain digestion solution at 37 °C for 5 min. After digestion, the suspension was filtered through a 70 µm cell strainer to remove any remaining undigested tissue. The cell suspension was centrifuged at 300 g for 5 min, and the cell pellet was resuspended in 1 mL of DPBS to generate a single‐cell suspension. For cell surface marker staining, the cells were incubated with PE/Cyanine7 anti‐mouse CD133 antibody (Biolegend, Catalog #141209) and FITC anti‐mouse CD24 antibody (Biolegend, Catalog #101806) on ice for 30 min, according to the recommended concentrations. After incubation, the cells were washed twice with DPBS and resuspended in 1 mL of DPBS. The cell population was then sorted using a BD FACSAria Fusion Flow Cytometer, and the data were analyzed using FlowJo software.

### Intracerebroventricular Injection and In‐Utero Electroporation (IUE)

The target plasmids or chemical inhibitors were mixed in proportion to the concentration and inhaled into beveled glass micropipettes for use. Sterilize bench and instrument. Timed pregnant ICR mice were anesthetized with 2,2,2‐tribromoethanol (Sigma, Cat# T48402). Cut the medioventral line, expose the uterine, find the fetal cerebral cortex, and inject the mixture into the lateral ventricle. For electroporation, the positive electrode was attached to the side of the target cortex. Five 50 ms pulses of 35–40 V with a 950 ms inter‐pulse interval were delivered with the tweezer electrodes connected to the electroporator (BTX ECM830).

### Single‐Cell RNA Sequencing Analysis

Single‐cell sequencing data were analyzed from the E13 developmental stage using the publicly available database (GEO SuperSeries GSE153164) and processed with the Seurat R package.^[^ ^7^ ^]^ To begin with, DoubletFinder was employed to remove doublet cells. Quality control parameters were set to exclude cells with less than 10% mitochondrial reads and more than 500 detected genes. This step ensured the retention of high‐quality cells for further analysis. The “FindVariableFeatures” function was used to select variable genes, followed by Principal Component Analysis (PCA) for data reduction. Based on the PCA results, cell clustering was achieved using the “FindNeighbors” and “FindClusters” functions. For Gene Pathway Scoring, major cell types were identified by their characteristic gene expression profiles. To calculate pathway scores for specific gene sets, Seurat's `AddModuleScore` function was used for each cell and was computed based on the gene list for selected terms, and the average value was taken as the final score for each cell type.

### Co‐Immunoprecipitation (Co‐IP)

Mouse IFITM2 and PTK2 cDNA were obtained by polymerase chain reaction (PCR). *Ifitm2* cloned into pCAG‐3HA to generate pCAG‐HA‐ *Ifitm2* and *Ptk2* cDNA cloned into pCAG‐3FLAG to generate pCAG‐FLAG‐ *Ptk2*. The target plasmids were co‐transfected into 293T cells, and the plasmid with the HA label was transferred as a negative control. After two days of cell culture, the protein was harvested. Resuspend the cells in 200 µL of IP lysis buffer. Sonicate cells 4–5 times for 5 s and place them directly on ice. Take out 20 µL cell lysate as an Input group and boil them. FLAG beads were added to the cell lysate and incubated at 4 °C overnight. The next day, the supernatant was discarded, and keep the beads for immunoprecipitation. Wash the beads with PBS and resuspend the beads in 200 µL RIPA buffer. In the last, boil them for 10 min in boiled water. The samples were stored in −80 °C refrigerator.

### Western Blot

Tissues and cells were lysed in RIPA lysis buffer with 1% Cocktail and 1% PMSF. Sonicate the lysis 4–5 times for 5 s, centrifuge at 8000 rpm for 10 min, and aspirate the supernatant. The lysate was added loading buffer and boiled for 10 min. The samples were loaded on 10% or 12% SDS‐PAGE. After transferring onto PVDF membranes and blocking with 5% milk (dissolved in PBST‐tween20), the membrane blots were incubated with primary antibodies at 4 °C overnight. The next day, the membranes were washed 3 times by PBST‐tween20 and incubated with the secondary antibodies at room temperature for 1 h. Finally, the membranes were washed 3 times with PBST‐tween 20. Develop and analyze by the imaging system (Odyssey, LI‐COR).

### RNA Sequencing

The samples were isolated from E12.5 wild‐type and *Ifitm2* cKO. Use the TRIzol reagent to extract total RNAs. The quality of RNA‐seq libraries was evaluated by Agilent 2100 Bioanalyzer and sequenced on an Illumina platform by Annoroad Gene Tech. (Beijing, China) Co. Ltd. Data deposited at GEO accession: GSE247448. GO analysis was performed using Metascape (http://metascape.org).

### Quantitative Real‐Time PCR

RNA was extracted using a TRIzol reagent. The purified RNA was reverse‐transcribed into cDNA using the First‐Strand cDNA Synthesis Kit (Tiangen, Cat# KR106‐02). Quantitative RT‐PCR was performed with the SuperReal PreMix Plus (SYBR Green) Kit (Tiangen, Cat# FP205‐03) on an RT‐PCR Detection System (LI‐COR Biosciences). GAPDH served as the internal control for normalizing mRNA levels. The primers are shown in Table S1 (Supporting Information).

### In Vitro Neurosphere Culture

Neural progenitor cells were isolated from the cortex of wild‐type and *Ifitm2* cKO of the E12.5 period. Cells were cultured in the medium containing DMEM/F12 (Gibco, Cat# 11330‐032), GlutaMax supplement (Gibco, Cat# 35050061), B‐27 supplement (1x, Thermo Fisher, Cat# 12587010), bFGF (5ng mL^−1^, Thermo Fisher, Cat# 13256029), EGF (5ng mL^−1^, Thermo Fisher, Cat# PHG0311) for 7 days, and changed the medium every 2 days.

### Quantification and Statistical Analysis

The fluorescence intensity of confocal microscopy images was assessed using either a Zeiss LSM 880 with Airyscan or a Leica Stellaris microsystem. To ensure comparability across groups within experiments, consistent settings were maintained, including laser power, pinhole size, and gain. Images were processed and analyzed using ImageJ software (NIH, USA). Regions of interest (ROIs) were delineated on each image to measure fluorescence intensity specifically. The “Measure” function in ImageJ quantified the mean fluorescence intensity within these ROIs, with background fluorescence subtracted from areas lacking specific staining to yield corrected fluorescence values. Fluorescence data were normalized by setting the mean intensity of the wild‐type group as 100%, with KO group intensities expressed relative to this benchmark. Statistical significance between groups was determined using Student's *t*‐test. For the quantification of fluorescence intensity distribution along the *Y*‐axis, rectangles of 200 µm height by 50 µm width were marked on selected images. The “Plot Profile” function from the “Analyze” menu generated plots of fluorescence intensity along the *Y*‐axis, which were used to evaluate distribution characteristics. These data were subsequently exported for further analysis. ImageJ 2.0.0 software facilitated the analysis of immunofluorescence co‐localization, area statistics, and intensity measurements, while Prism 9.4.1 software was employed for statistical analysis. Parameters and repetition counts were detailed in the figure legends. A *p*‐value of <0.05 was deemed statistically significant in all analyses.

## Conflict of Interest

The authors declare no conflict of interest.

## Author Contributions

Y.L., W.Z., and L.L. These authors contributed equally to this work Y.L. and W.Z. conceived the project, J.J. and J.P. supervised the project and acquired the funding support, Y.L., W.Z., L.L., and J.L. performed the experiments, W.Z. and S.Z. analyzed the high‐throughput sequencing data. Y.L. and W.Z. wrote this manuscript with input from all authors.

## Supporting information



Supporting Information

## Data Availability

Research data are not shared.
