# Supplementary material for: IFITM2 Modulates Endocytosis Maintaining Neural Stem Cells in Developing Neocortex
Source: Adv Sci (Weinh). 2025 Mar 7;12(17):2501593. doi: 10.1002/advs.202501593 (PMC12061285; doi:10.1002/advs.202501593)
Supplement: Supplementary file 1 — Supporting Information [file ADVS-12-2501593-s001.pdf]

ADVANCED SCIENCE
Open Access

Supporting Information

for *Adv. Sci.*, DOI 10.1002/advs.202501593

IFITM2 Modulates Endocytosis Maintaining Neural Stem Cells in Developing Neocortex

Yuqing Lv, Wenzheng Zou, Lin Li, Shukui Zhang, Jiaqi Liang, Jiali Pu and Jianwei Jiao**

Supporting Information:

IFITM2 Modulates Endocytosis Maintaining Neural Stem Cells in Developing Neocortex

Yuqing Lv[1,2,3,7], Wenzheng Zou[1,2,3,4,7], Lin Li[1,2,3,7], Shukui Zhang[1,2,3], Jiaqi Liang[1,2,3], Jiali Pu[6,*], Jianwei Jiao[1,2,3,5,*]

This file includes:

Figure S1-S7

Table S1

Figure S1

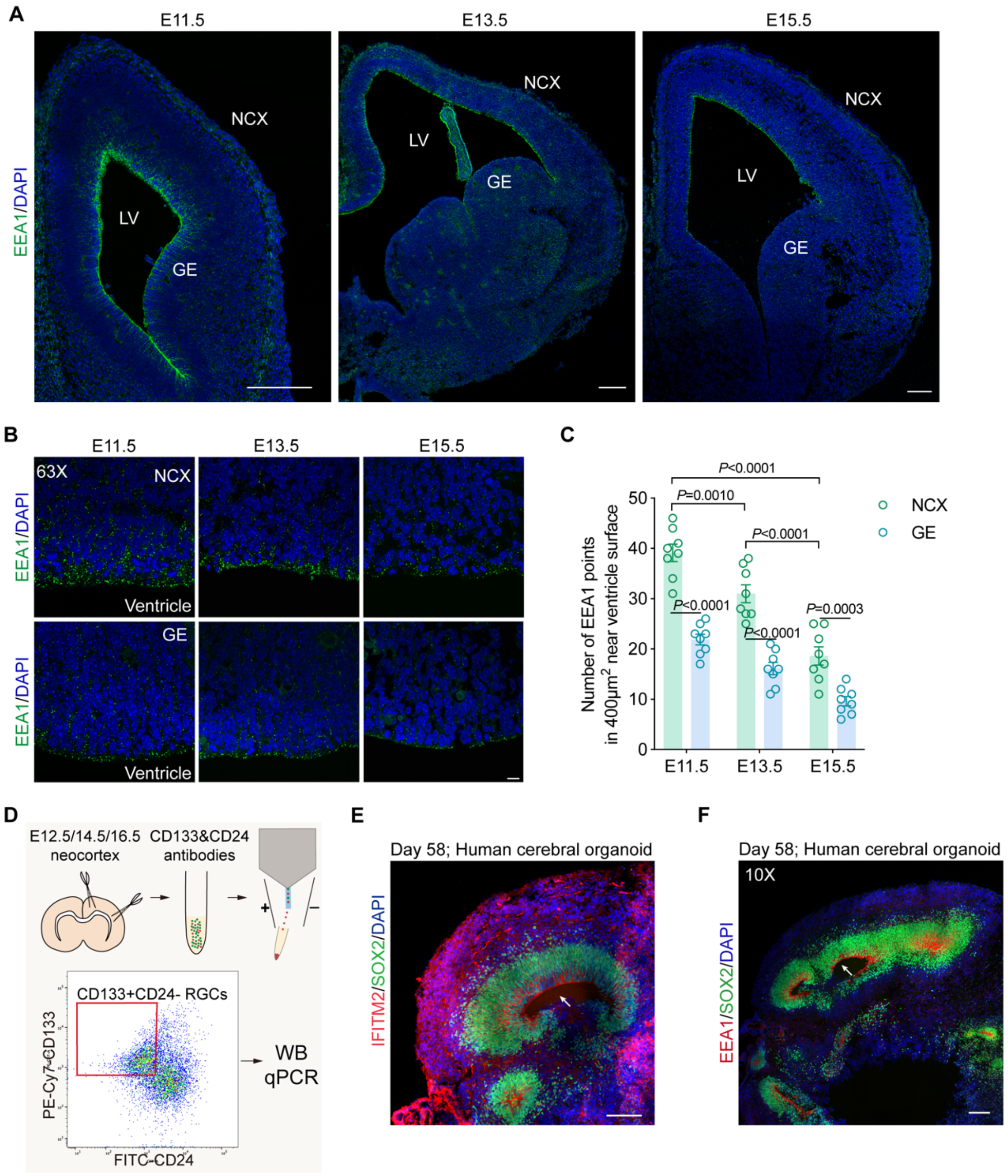

Figure S1. Decline in endosomes along the ventricular surface from early to late neurogenesis in brain neocortex.

(A) Representative images from brain sections at E11.5, E13.5, and E15.5, stained for EEA1 (early endosome antigen 1, green), and DAPI (blue). Images were acquired using a 20X objective lens. NCX, neocortex; GE ganglionic eminence. Scale bars, 200 μm.

(B) Representative images for NCX and GE regions stained for EEA1 (early endosome antigen 1, green), and DAPI (blue). Images were acquired using a 63X objective lens. Scale bar, 10 μm.

(C) Quantification of EEA1 positive early endosomes per 400 μm^2. Data are shown as mean±s.e.m. for each time point (E11, E13, E15; *n*=8 area from 4 brains per time point). Two-way ANOVA follow by Tukey's multiple comparisons test.

(D) Schematic diagram illustrating the use of flow cytometry to isolate CD133+CD24- RGCs from E12.5, E14.5, and E16.5 neocortex.

(E) Representative images of day 58 human cerebral organoid immunostaining with IFITM2 (red), SOX2 (green), and DAPI (blue). The white arrow indicates the ventricle-like structure. Scale bar, 100 μm.

(F) Representative images of day 58 human cerebral organoid immunostaining with EEA1 (red), SOX2 (green), and DAPI (blue). The white arrow indicates the ventricle-like structure. Scale bar, 100 μm.

Figure S2

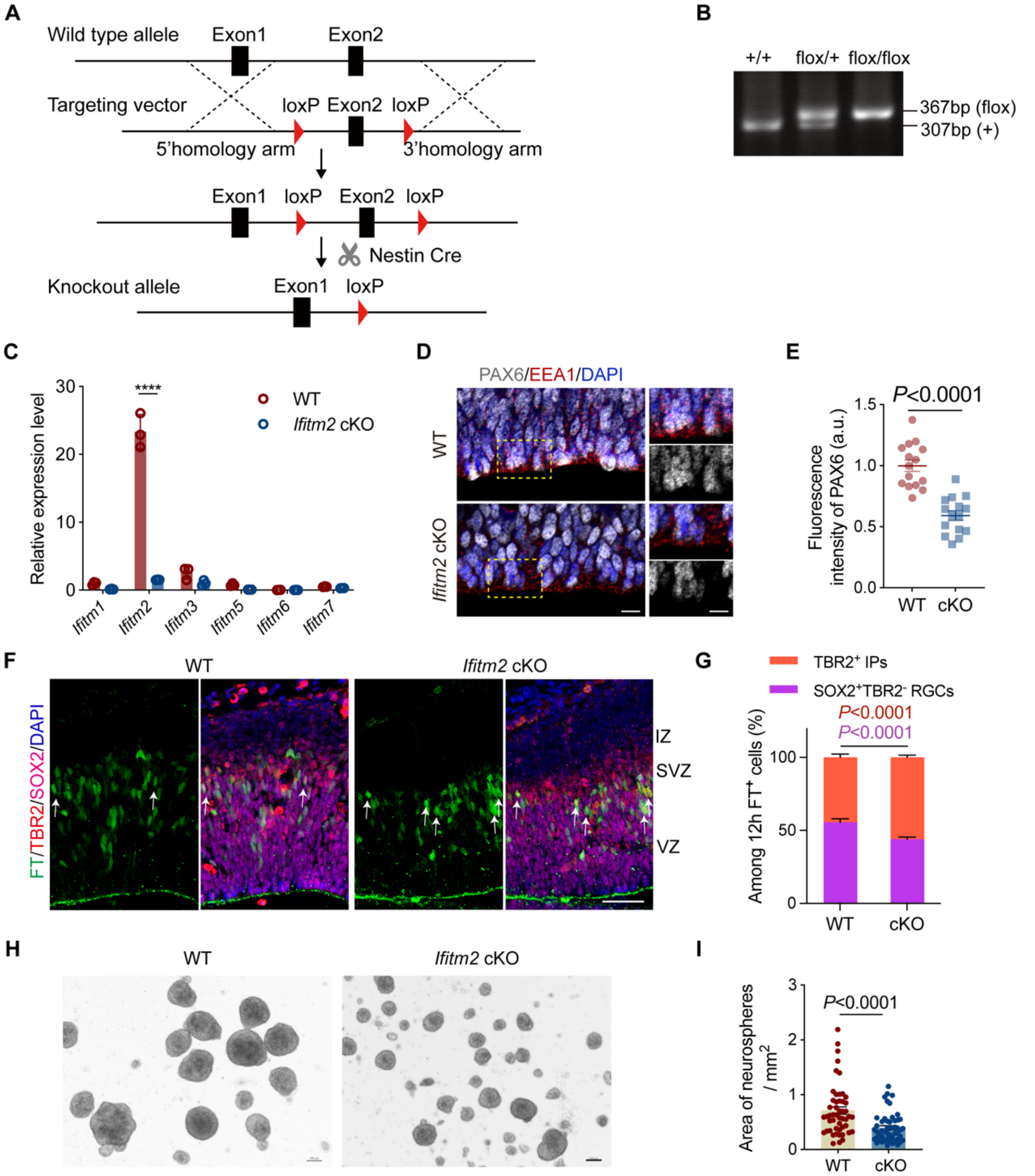

Figure S2. IFITM2 deletion enhances differentiation of cortical RGCs.

(A) Schematic diagram of the generation of the *Ifitm2* cKO mouse using the CRISPR-Cas9 strategy.

(B) Representative image of genomic PCR identification at the flox sites.

(C) Q-PCR analysis of *Ifitm* family genes expression from the E11.5 wild-type and *Ifitm2* cKO mouse brains (n=3 independent experiments). All Ct values were normalized to *Gapdh* control. Data are shown as mean ± s.e.m, ***$P < 0.0001$. Two-tailed unpaired Students' t-test.

(D) Representative images of E13.5 wild-type and *Ifitm2* cKO cerebral cortex sections stained with PAX6 (white), EEA1 (red) and DAPI (blue). Scale bars, 10 μm (left) and 5 μm (right).

(E) Quantification of PAX6 fluorescence intensity in cells located on the surface of the brain ventricle (n= 15 cells, from two embryos in each group). Data are shown as mean ± s.e.m. Two-tailed unpaired Students' t-test.

(F) Representative images of FlashTag-labeled (green) daughter cells 12 hours after RGC division, stained with SOX2 (magenta), TBR2 (red), and DAPI (blue). White arrows indicate $FT^{+}TBR2^{+}$ daughter cells. Scale bar, 50 μm.

(G) Quantifications of the percentage of $TBR2^{+}$ cells and $SOX2^{+}TBR2^{-}$ cells among FT^{+} cells 12 hours after FT labeling from four biological replicate experiments. Data are shown as mean + s.e.m. Two-way ANOVA followed by Tukey's post-hoc test.

(H) Representative images of neurospheres of wild-type and *Ifitm2* cKO cultured for 5 days. Primary neural stem cells were isolated from E12.5 cortices and cultured in suspension culture dishes. Scale bar, 100 μm.

(I) Quantification of the area of neurospheres (n=50 neurospheres per group). Data are shown as mean ± s.e.m. Two-tailed unpaired Students' t-test.

Figure S3

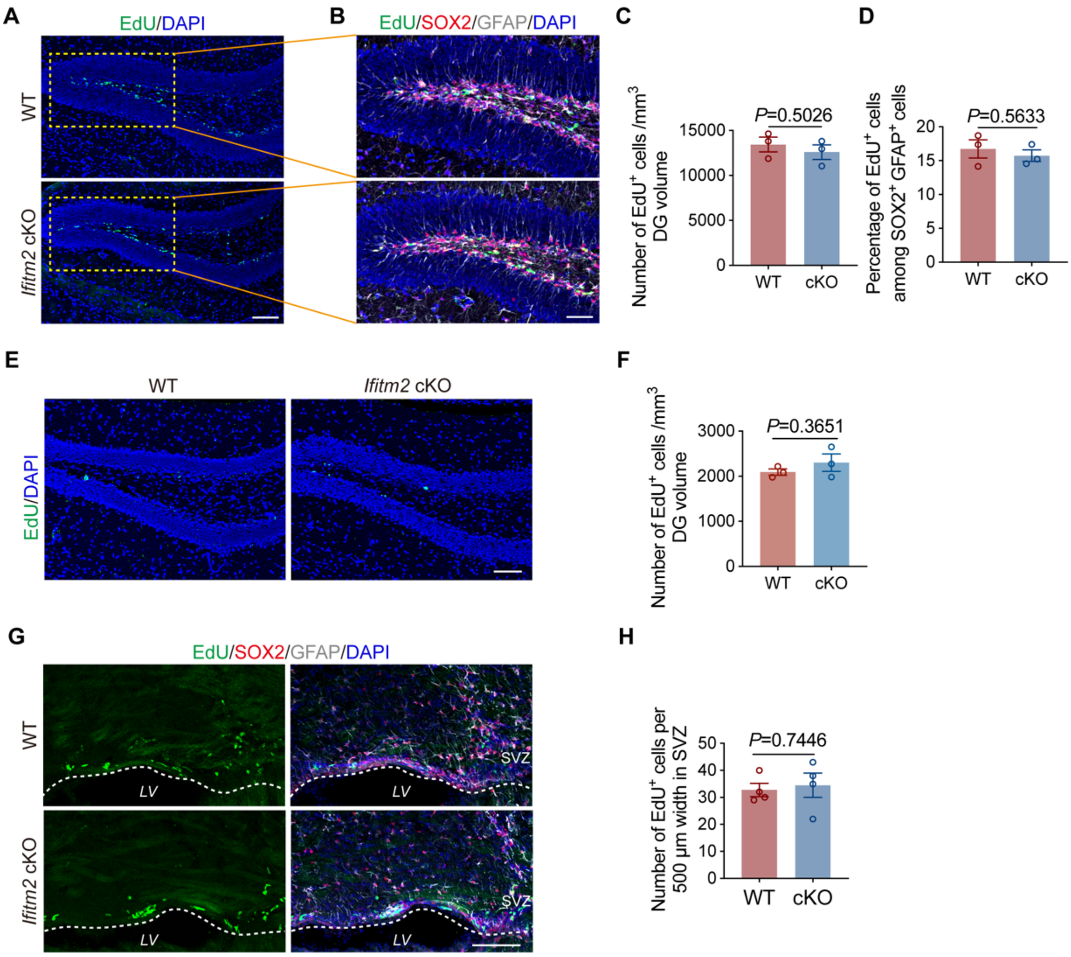

Figure S3. IFITM2 deletion does not significant affect adult neurogenesis.

(A-B) Representative images of EdU labeling in the dentate gyrus (DG) region of the P21 mouse hippocampus after a 24-hour injection, with staining for EdU (green), SOX2 (red), GFAP (grey), and DAPI (blue). Scale bar, 100 μm (A) and 50 μm (B).

(C-D) Quantification of the number of EdU^+ cells per 1 mm^3 in the DG region (C) and the percentage of EdU^+ cells among $SOX2^+GFAP^+$ cells (D) (*n*=3 brains per group). Data are shown as mean ± s.e.m. Two-tailed unpaired Student's *t*-test was used.

(E) Representative images of EdU labeling in the DG region of the adult mice (2 month aged) hippocampus after 24-hour injection. Scale bar, 100 μm.

(F) Quantification of the number of EdU^+ cells per 1mm^3 in DG region (*n*=3 brains per group). Data are shown as mean ± s.e.m. Two-tailed unpaired Students' t-test was used.

(G) Representative images of EdU labeling in the SVZ region of the adult mice neocortex after 24-hour injection, and stained with EdU (green), SOX2 (red), GFAP (grey), and DAPI (blue). Scale bar, 100 μm.

(H) Quantification of the number of EdU^+ cells per 500μm column in dorsal cortex (*n*=4 brains per group). Data are shown as mean ± s.e.m. Two-tailed unpaired Students' *t*-test was used.

Figure S4

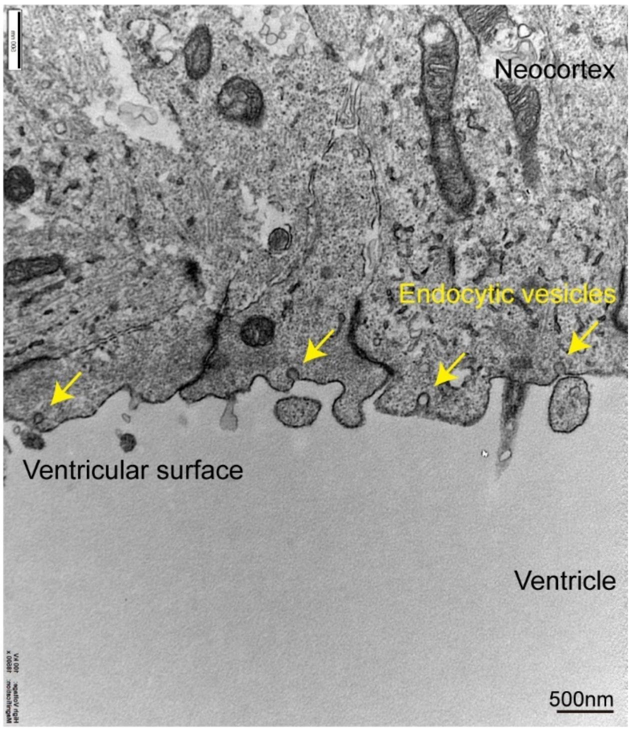

Figure S4. Representative TEM image of the E14.5 cortical ventricular surface. The yellow arrows indicate the endocytic vesicles on the apical side of RGCs. Scale bar, 500 nm.

Figure S5

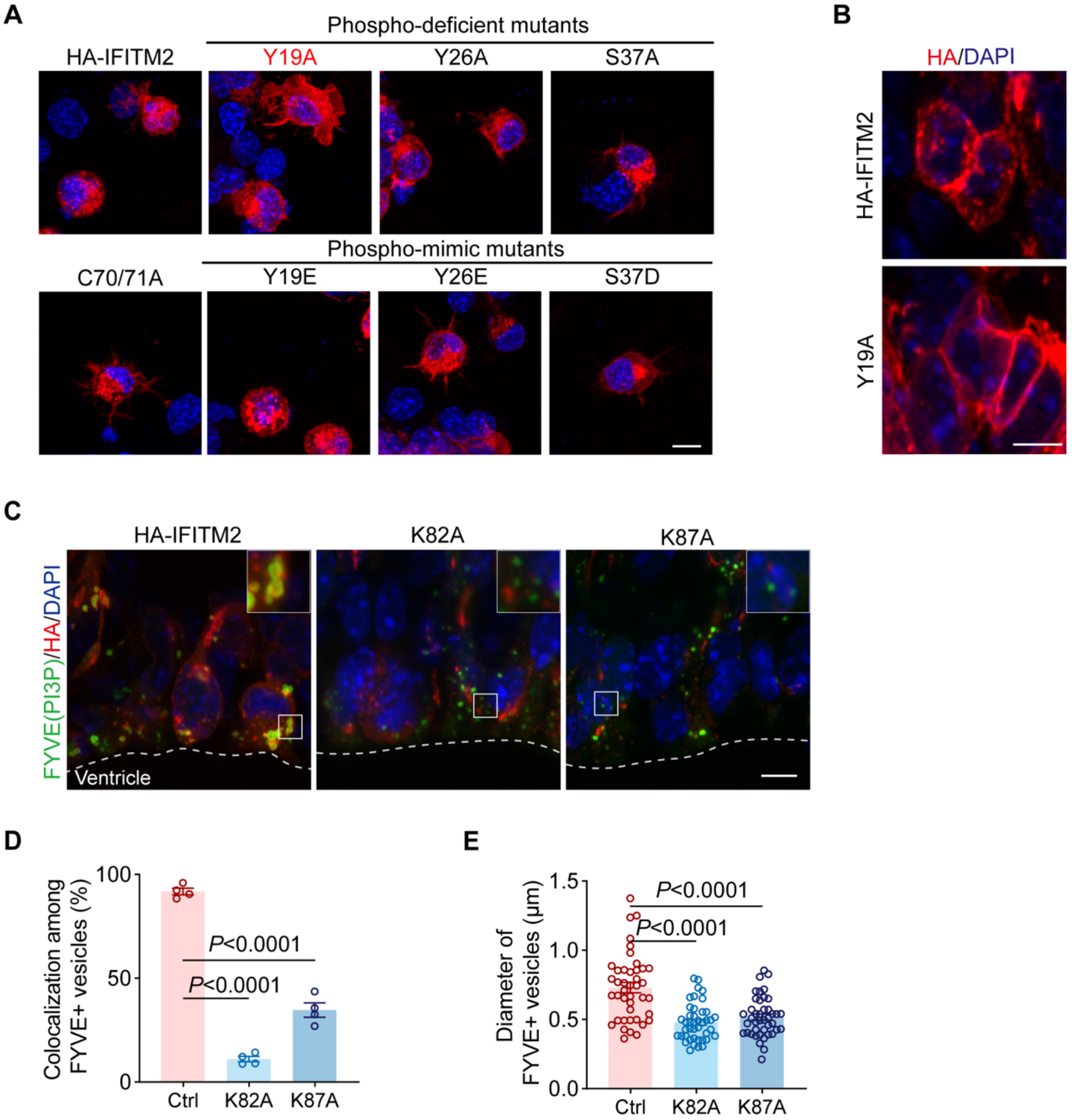

Figure S5. Functional analysis of IFITM2 mutants.

(A) Representative images of the Neuro-2a cells transfected with HA-IFITM2, Y19A, Y26A, S37A, Y19E, Y26E, S37D, or C70/71A mutants. The cells were stained with HA (red) and DAPI (blue). Scale bar, 10μm.

(B) Representative *in-utero* electroporation (IUE) images of the RGCs electroporated with HA-IFITM2 and HA-IFITM2-Y19A. Sections were stained with HA (red) and DAPI (blue). Scale bar, 10 μm.

(C) Representative IUE images of the neocortex electroporated plasmids encoding GFP-2×FYVE (green) and HA-IFITM2, HA-IFITM2-K82A, or HA-IFITM2-K87A, stained with HA (red) and DAPI (blue). Scale bar, 5 μm.

(D) Quantification of the percentage of GFP-2×FYVE$^+$ vesicles that co-localize with HA-IFITM2 or its K82A, K87A mutants, relative to all GFP-2×FYVE$^+$ vesicles from 900 μm^2 area near the ventricle surface (*n*=4 brains per group). Data are shown as mean ± s.e.m. Two-tailed unpaired Students' *t*-test.

(E) Quantification of the diameter of GFP-2×FYVE$^+$ vesicles in RGCs electroporated with HA-IFITM2 or its K82A, K87A mutants (*n*=42 vesicles from 4 brains per group). Data are shown as mean ± s.e.m. Two-tailed unpaired Students' *t*-test.

Figure S6

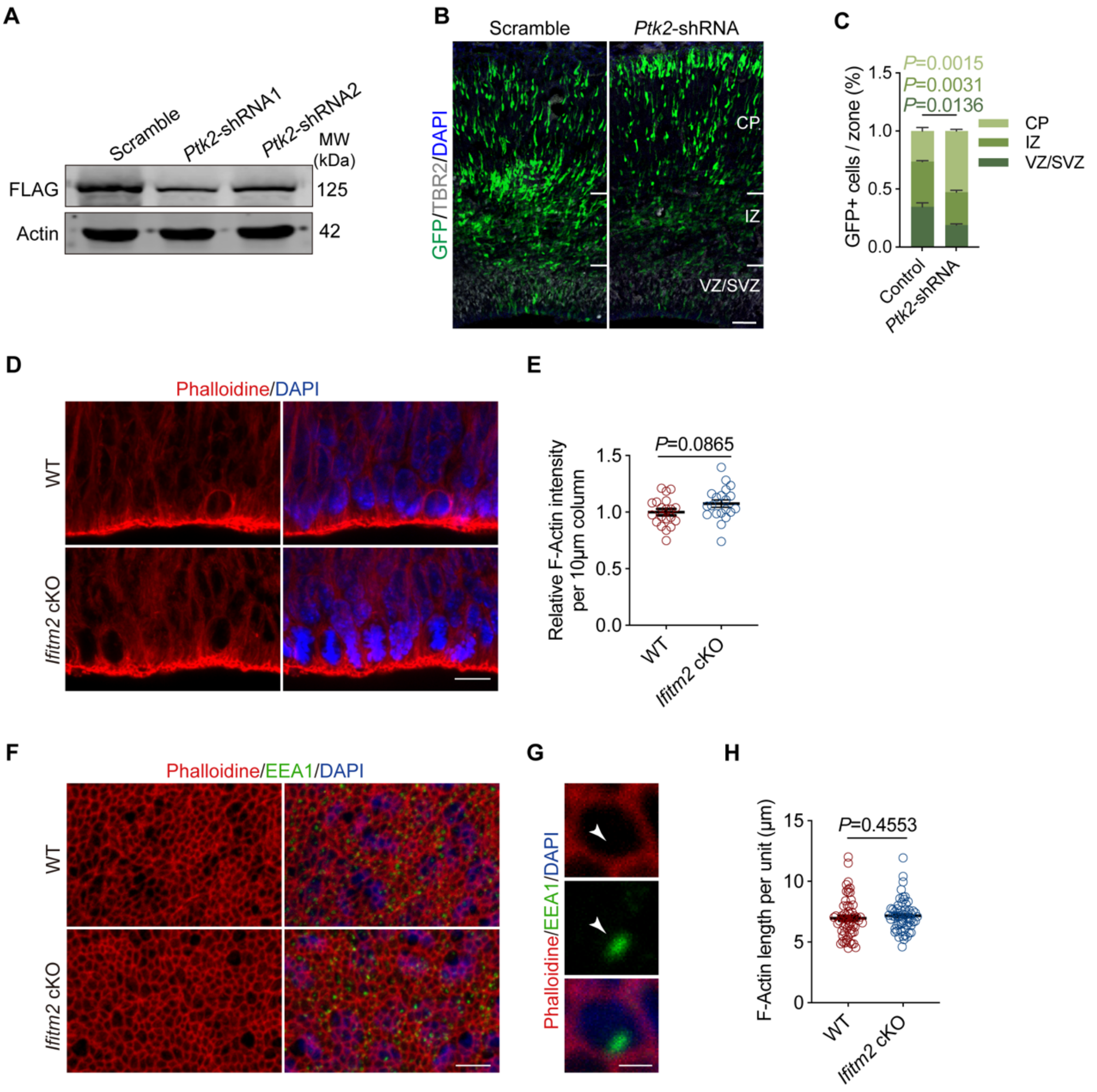

Figure S6. FAK inactivation promotes neural differentiation.

(A) Western blot analysis of Flag-tagged PTK2 levels after transfecting the plasmids Scramble RNA, *Ptk2*-shRNA1, or *Ptk2*-shRNA2 in HEK293T cells to verify the knockdown efficiency.

(B) Representative *in-utero* electroporation (IUE) images of the neocortex electroporated with GFP reporter and Scramble shRNA or *Ptk2*-shRNA1 at E14.5 and analyzed at E16.5. Sections were stained with TBR2 (gray) and DAPI (blue). Scale bar, 50 μm.

(C) Quantification of the GFP$^+$ cells in the VZ/SVZ, IZ, and CP regions per 200 μm column (*n*=3 brains per group). Data are shown as mean + s.e.m. Two-way ANOVA followed by Tukey's post-hoc test.

(D) Representative images of E13.5 wild-type and *Ifitm2* cKO in the VZ region of cortical coronal section stained for Phalloidine (F-actin, red) and DAPI (blue). Scale bar, 10 μm.

(E) Quantification of the intensity of F-actin per 10 μm column (*n*=20 from 3 brains per groups).

(F) Representative en face images of E13.5 wild-type and *Ifitm2* cKO ventricular zone surface stained for Phalloidine (F-actin, red), EEA1 (green), and DAPI (blue). Scale bar, 10 μm.

(G) Magnified images showed the localization of EEA1 and F-actin in the unit at the ventricular zone surface. Scale bar, 1 μm.

(H) Quantification of the perimeter of individual junctions (*n*=60 units from 3 brains per groups). Data are shown as mean ± s.e.m. Two-tailed unpaired Students' t-test was used.

Figure S7

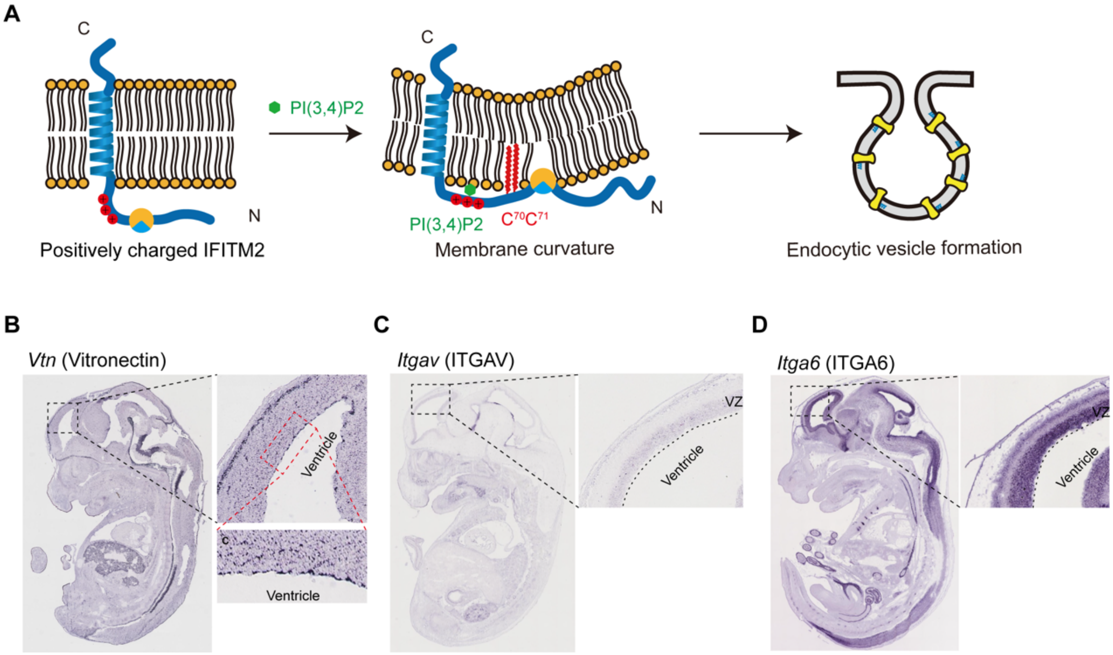

Figure S7. Predicted working model of IFITM2-mediated membrane curvature.

(A) The interaction of IFITM2 with phosphoinositide, the palmitoylation of C70 & 71 residues, and the insertion of amphipathic helix into the lipid bilayer together induce a negative membrane curvature.

(B-D) Expression patterns of *Vtn* (B), *Itgav* (C), *Itga6* (D) in sagittal sections of embryo at E14.5 (*in situ* hybridization maps from www.genepaint.org) [28].

Table S1. Primers for qRT-PCR.

IFITM1 RT1-F	CTGACCATGTGGTCTGGTCC
IFITM1 RT1-R	GCCGTGAGGATGGTGAAGAA
IFITM1 RT2-F	CTGAGATCTCCACGCCTGAC
IFITM1 RT2-R	GGAGCTGATGTTCAGGCACT
IFITM2 RT1-F	CTTCTTGTCCACCAATGCCG
IFITM2 RT1-R	AGCAGGCGTTGAAGAAGAGT
IFITM2 RT2-F	AGGAGTACGGGGTGACTGAG
IFITM2 RT2-R	CCCACCATCTTCCTGTCCCT
IFITM3 RT1-F	GAGGACAGCCCCCAAACTAC
IFITM3 RT1-R	TCACGGAGTAGGCATAGGCT
IFITM3 RT2-F	GACAGCCCCCAAACTACGAA
IFITM3 RT2-R	CCGATCCCTAGACTTCACGG
IFITM5 RT1-F	CCTCTCCATGGGAACACCTG
IFITM5 RT1-R	GTAGCACTTGGCTTTGGAGC
IFITM5 RT2-F	GCTGGTCCACTCTGTCAAGG
IFITM5 RT2-R	GCCGCAGAGTCTTTGGCTA
IFITM6 RT1-F	CCGGATCACATTACCTGGTCT
IFITM6 RT1-R	GATGAGGGAGATGACCAGAGC
IFITM6 RT2-F	CAGCCGGATCACATTACCTGG
IFITM6 RT2-R	ATGAGGGAGATGACCAGAGCA
IFITM7 RT1-F	CCTGAGATCTCCAAGCCTGAT
IFITM7 RT1-R	ACCATGACGACGGAGAGGAT
IFITM7 RT2-F	TGTGGTCTGGTCTCTGTTCAA
IFITM7 RT2-R	AGGATGACCATGACGACGGA
